# Aptamer Engineering: Strategies for Discovering Functional Nucleic Acids for Next‐Generation Diagnostics and Biosensing

**DOI:** 10.1002/advs.202516852

**Published:** 2025-11-26

**Authors:** John V. L. Nguyen, Ahlem Meziadi, Cyrill Brunner, Maureen McKeague, Lidija Malic, Maryam Tabrizian

**Affiliations:** ^1^ Department of Biomedical Engineering McGill University 3775 University Street Montreal QC H3A 2B4 Canada; ^2^ Bruker Switzerland AG Industriestrasse 26 Faellanden CH‐8117 Switzerland; ^3^ Department of Chemistry McGill University 801 Sherbrooke Street West Montreal QC H3A 0B8 Canada; ^4^ Department of Pharmacology and Therapeutics McGill University 3655 Promenade Sir‐William‐Osler Montreal QC H3G 1Y6 Canada; ^5^ Life Sciences Division National Research Council of Canada 75 de Mortagne Boulevard Boucherville QC J4B 6Y4 Canada; ^6^ Faculty of Dental Medicine and Oral Health Sciences McGill University 2001 McGill College Avenue Montreal QC H3A 1G1 Canada

**Keywords:** antibody mimetics, aptasensors, biosensors, directed evolution, in vitro selection, molecular engineering, nucleic acid engineering

## Abstract

Aptamers are highly selective nucleic acid ligands that overcome many challenges of antibodies, including structural instability and high production costs. The implementation of aptamers into routine research and commercial applications has advanced steadily, though it has been tempered by the limitations of existing discovery methods, which remain relatively slow and low‐throughput. SELEX, the gold‐standard method for aptamer discovery, has played a pivotal role in the field. As the demand for faster and more tailored solutions grows, there is increasing recognition of the value of newer approaches that can expedite the discovery and optimization of aptamers for specific applications. This review discusses recent advances in the methods used for aptamer engineering, including both wet lab methods performed in vitro, and dry lab methods performed in silico. Applications of engineered aptamers described in recent literature and discuss new developments that could further lead to innovative new applications of aptamers for use both within and outside of the laboratory are discussed. Within the laboratory, these applications include (but are not limited to) target‐binding assays and integration into analytical nanomaterials, and outside of the laboratory, diagnostics and biosensing, which will be discussed at length.

## Graphical abstract

The advent of aptamers has highlighted their potential as alternatives to antibodies, overcoming limitations of structural instability and production cost. However, conventional approaches such as SELEX remain slow and labor‐intensive. This review examines recent advances in aptamer engineering, emphasizing in vitro and AI‐driven in silico strategies, and explores emerging applications that may broaden their translational impact.

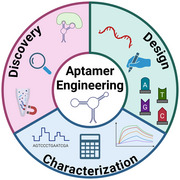

## 1 Introduction

Nucleic acids (DNA and RNA), molecules which were classically only thought of as the medium for encoding genetic information,^[^ ^1^ ^]^ have since been proven to possess an intrinsic ability to bind to other molecules. Indeed, many DNA or RNA motifs interact with proteins such as polymerases and transcription factors. However, aptamers are polymer sequences, typically single‐stranded DNA or RNA, that fold into 3D structures and have the ability to bind to a range of molecules with high affinity and specificity via intermolecular forces. Since their discovery,^[^ ^2^, ^3^ ^]^ the premise that aptamers could one day be applied to a magnitude of different problems both within and outside of the laboratory has been a key motivation for further study of these biomolecules. While an occurrence of a naturally produced aptamer can be observed by the ligand‐binding element of riboswitches,^[^ ^4^ ^]^ the production of aptamers today is largely an in vitro process.

The ability of aptamers to bind to specific targets has led to comparisons with antibodies.^[^ ^5^ ^]^ Their typical size range of 20 to 100 nucleotides^[^ ^6^ ^]^ means that they are smaller in size compared to antibodies, allowing for improved transport, tissue penetration,^[^ ^7^ ^]^ and small particle sensing.^[^ ^8^ ^]^ Their nucleic acid composition makes them faster, easier, and less expensive to synthesize than peptides^[^ ^7^ ^]^ and also more shelf‐stable than antibodies.^[^ ^9^ ^]^ Their screening process means that aptamers can be found for non‐immunogenic molecules and against toxins that would otherwise be lethal to animals during antibody production.^[^ ^10^ ^]^ Their high selectivity means that they are less likely to give false positive results, an issue that has been plaguing antibodies.^[^ ^11^ ^]^ However, their nucleic acid structure can also be detrimental to potential applications, as aptamers composed of unmodified nucleotides are quickly degraded by nucleases and have half‐lives as short as two minutes in vivo.^[^ ^12^ ^]^ Their screening process also serves as one of the most substantial reasons why aptamer adoption has been slow, as current techniques in aptamer screening have only currently yielded two United States Food and Drug Administration approved therapeutic aptamers, pegaptanib (brand name Macugen)^[^ ^13^ ^]^ and avacincaptad pegol (brand name Izervay),^[^ ^14^ ^]^ and the UTexas Aptamer Database, which is currently the largest functioning online database of aptamers,^[^ ^15^ ^]^ presently only lists 1,551 known aptamers. Despite over thirty years since their discovery, observers have questioned why progress in discovering robust novel aptamers, up to this point, has been slow.^[^ ^16^ ^]^

Although nuclease degradation in vivo presents challenges for therapeutic use, aptamers offer strong potential in areas such as medical diagnostics and non‐medical sensing, where their properties can be particularly advantageous. The discovery of aptamers for these applications, including against toxins and small particles,^[^ ^17^, ^18^ ^]^ shows the superiority of aptamers not just in usual applications, but in niche areas where antibody generation is difficult. Further, the benefits of aptamers make them suitable for use in precision diagnostics, an emerging subset of precision medicine that tailors the diagnosis and treatment of disease to each individual patient. However, the current yield of suitable aptamers has been too low to be disruptive to these areas, and optimization of both the aptamer molecules and the aptamer discovery process is needed before we can realize a larger role of aptamers in practical applications.

**Figure 1:**
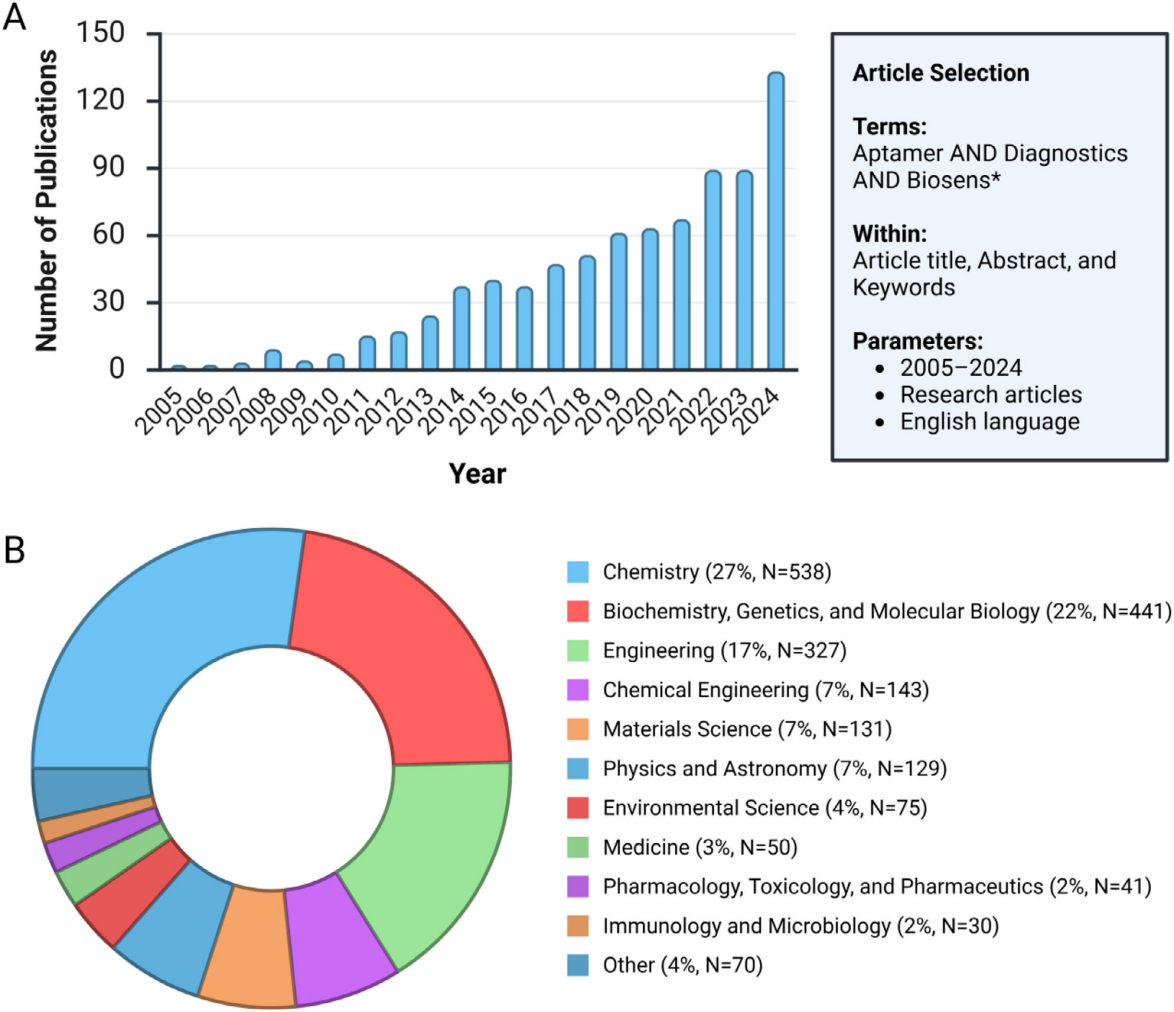
Quantitative metrics for English language research articles published containing the terms “aptamer” AND “diagnostics” AND “biosens*,” among the article title, abstract, and keywords, and published between the period from 2005 to 2024 inclusive, as indexed by Scopus as of November 1, 2025. A) Number of publications by year. B) Proportion of publications by research area.

**Figure 2:**
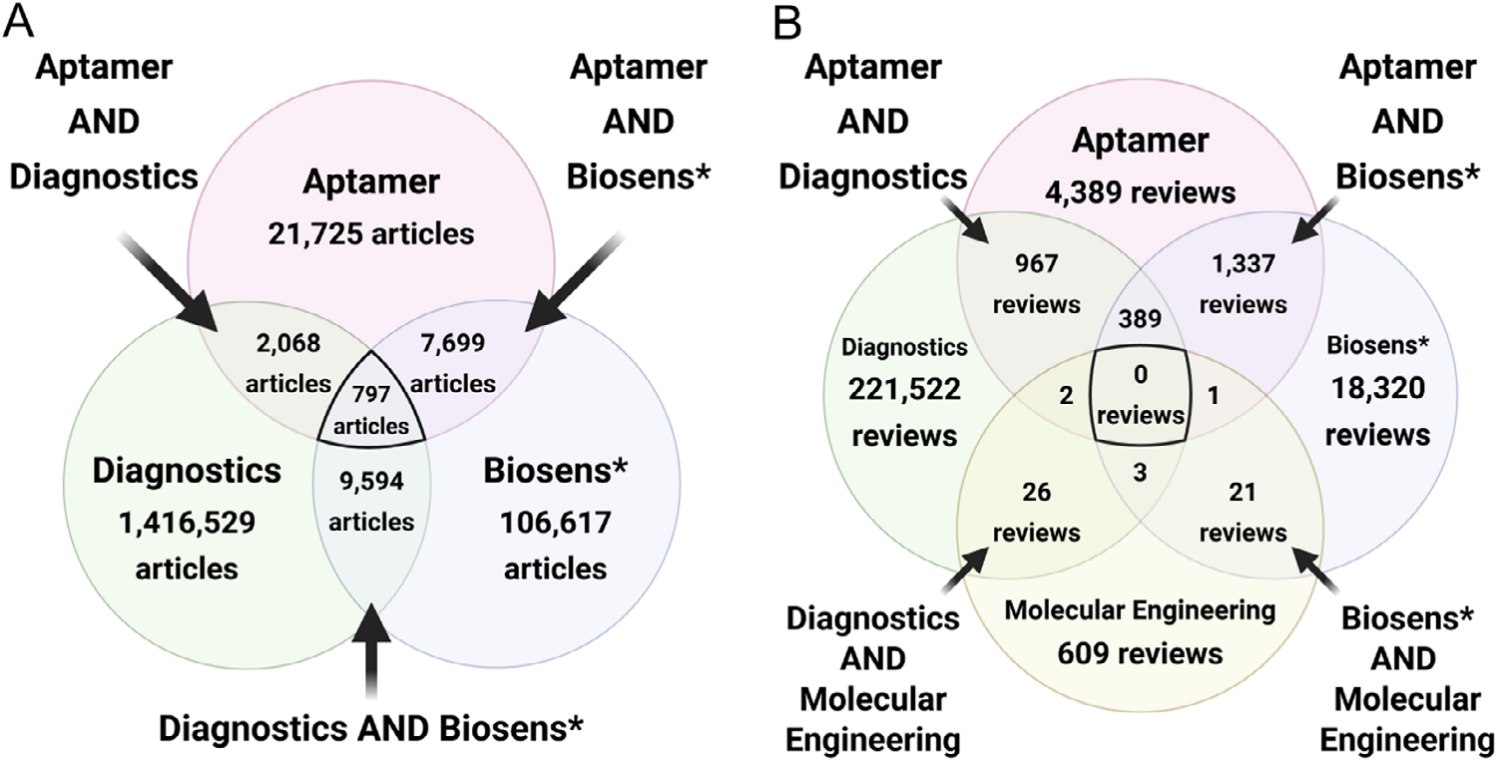
Number of English language articles per term(s) searched among the article title, abstract, and keywords, and published between the period from 2005 to 2024 inclusive, as indexed by Scopus as of November 1, 2025. A) Research articles containing the terms “aptamer” AND “diagnostics” AND “biosens*” and B) review articles containing the terms “aptamer” AND “diagnostics” AND “biosens*” AND “molecular engineering.”.

Nonetheless, research progress in this area has been gaining pace, especially within the last few years, and to quantitatively gauge the scholarly output and application areas, we performed a Scopus search for publications containing the terms “aptamer” AND “diagnostics” AND “biosens*” among the article title, abstract, and keywords, limited to the preceding 20‐year range of 2005 to 2024 inclusive, and limited the document type to research articles only (i.e., reviews, books/book chapters, conference papers, etc., were all excluded from the results). We observed a general trend of increasing publications year‐over‐year during most years for publications, which met these criteria, and saw that the subject area for these articles extended beyond the expected chemistry, biochemistry, genetics, and molecular biology, but also to areas including materials science, physics and astronomy, and environmental science, indicating the increasing interest in this area across a broad range of disciplines (**Figure** **1**), and a sizable collection of 797 research articles with these terms across disciplines (**Figure** **2**A).

The growing collection of recent literature in this area and the introduction of modern molecular engineering and nucleic acid engineering methods into the aptamer development process have led to the formation of a new subdiscipline of nucleic acid engineering that is aptamer engineering. While previous reviews have discussed engineering of a broad range of functional nucleic acids,^[^ ^19^, ^20^ ^]^ and nanoengineering incorporating nucleic acids,^[^ ^21^ ^]^ no review has focused on the engineering aspect of aptamers specifically for the purposes of diagnostics and non‐medical sensing. We confirmed this by repeating our Scopus search with the same search filters, except with the addition of including “molecular engineering” as an additional search term and limiting the document type to review articles only (Figure 2B). The result was zero previous reviews, demonstrating that despite the active research in this area, there is an absence of reviews covering this scope. The abundant number of research articles and absence of previous reviews in this area demonstrates the materialization of this new subdiscipline in the research landscape.

Although many years have passed since their discovery, aptamers and aptamer‐based sensors, or aptasensors, have yet to achieve widespread commercialization. Traditionally, aptamers were identified through the systematic evolution of ligands by exponential enrichment (SELEX) process, a time‐consuming method. Today, advances in molecular engineering and nucleic acid engineering, including chemical modifications, analytical instrumentation, and computational approaches such as artificial intelligence (AI), are transforming aptamer discovery. By significantly increasing throughput and efficiency, these methods may enable aptamer‐based technologies to play a central role in future diagnostic and biosensing applications. We refer to this emerging integration of advanced tools and strategies as next‐generation aptamers.

In this Review, we will examine the recent trends in how aptamers are designed and the techniques used to characterize them. Additionally, we will introduce the applications of these aptamers in medical diagnostics and non‐medical sensing applications. Although a large proportion of emerging literature regarding aptamers explores their therapeutic potential, which will be beyond the scope of this Review, we direct interested readers to other excellent reviews that have already covered the progress of aptamers for therapeutic applications.^[^ ^22^, ^23^, ^24^ ^]^

## 2 Aptamer Engineering

Molecular engineering is a multidisciplinary field that focuses on designing, analyzing, and synthesizing molecules to achieve specific functions or properties. It combines principles of chemistry, physics, biology, and engineering to manipulate molecules at the atomic and molecular levels. The field is crucial in developing innovative solutions across industries such as medicine, materials science, energy, and nanotechnology.

Aptamer engineering is the application of molecular engineering principles, strategies, and techniques specifically to nucleic acid aptamers; thus, aptamer engineering is an emerging new subfield under nucleic acid engineering and molecular engineering. Many recent papers apply molecular engineering techniques to aptamers^[^ ^25^, ^26^, ^27^, ^28^ ^]^ and unveil the potential for a wide range of applications of aptamers in biotechnology, diagnostics, and therapeutics. A feature and constraint of aptamer engineering is that most reported aptamers are composed of natural nucleic acids. The use of nucleic acids enables rapid synthesis and sequencing during discovery; however, their chemical nature can limit stability and interaction with certain targets compared to other molecular scaffolds. Nevertheless, aptamer engineering offers numerous opportunities to improve performance and tailor sequences for specific downstream applications. These efforts encompass optimization of the discovery process, rational design parameters, and molecular strategies for sequence refinement. Major aspects of aptamers that can be engineered and improved will be discussed in Section 4. However, before discussing those advanced topics, a brief introduction to the classical method of aptamer discovery is first required.

**Figure 3:**
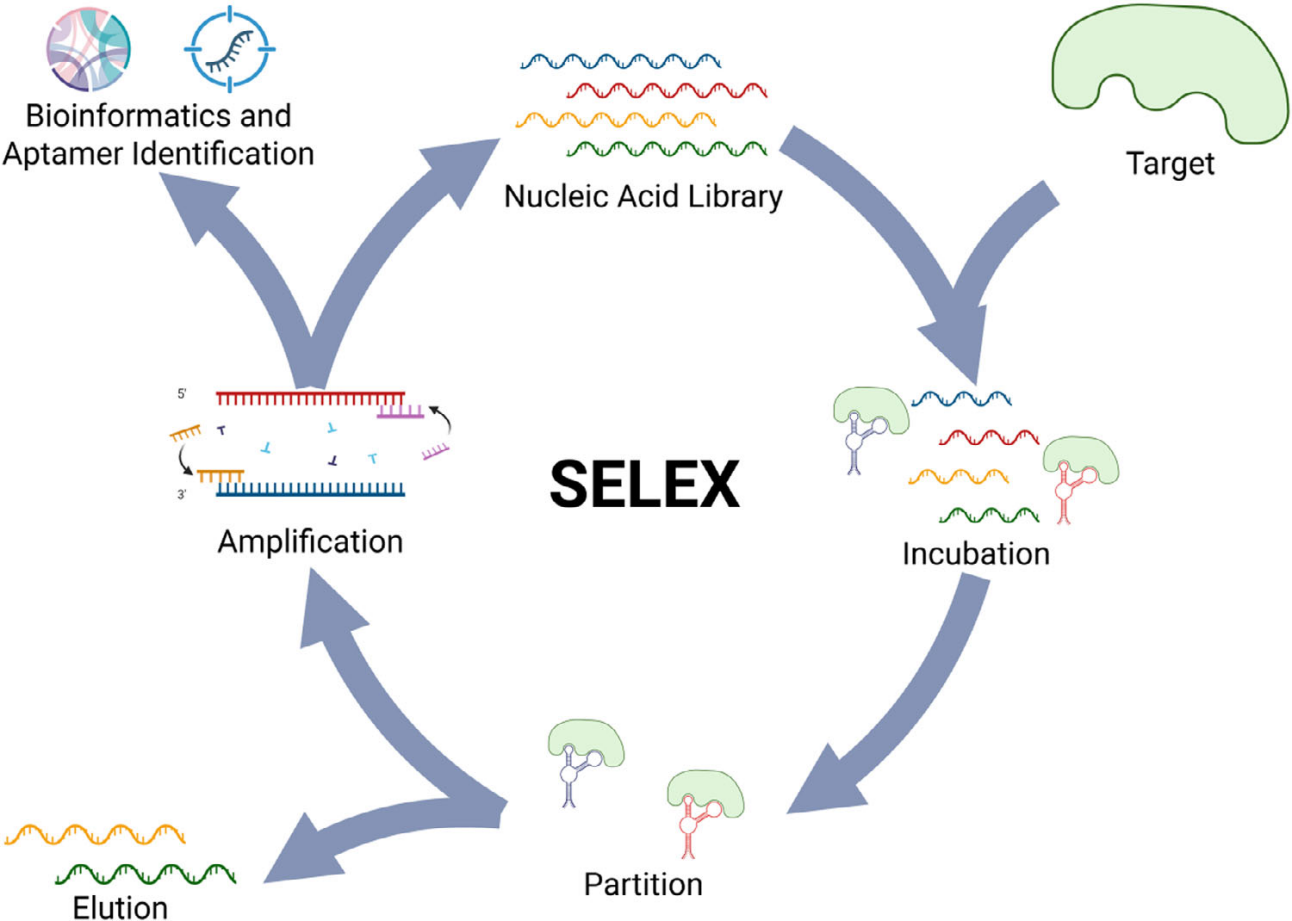
General outline of the SELEX process. A nucleic acid library is incubated with the target to facilitate binding, and the binders are partitioned from the non‐binders. In a positive selection step, the binders are amplified, and in either a counter‐selection or negative selection step, the non‐binders are amplified. The amplified nucleic acid enriches the library for potential aptamers and is used in the next round of SELEX. After several rounds of SELEX, sequencing and bioinformatics are used to identify the aptamer candidates.

The gold‐standard method of aptamer discovery is SELEX. The SELEX process involves the generation of a large single‐stranded oligonucleotide library or pool, in which oligonucleotide sequences are either fully or partially randomized. In a positive selection step, the oligonucleotide library is then incubated with the target, after which the unbound sequences are washed away, while the remaining bound sequences with affinity for the target are subsequently eluted and retained. Alternatively, or even additionally, a negative/counter selection step could take place, in which the oligonucleotide library is incubated with a non‐target, in which the unbound sequences without affinity for the non‐target are retained, while the bound sequences with affinity for the non‐target are discarded. Following incubation of the oligonucleotide library with either the target or non‐target, retained sequences are amplified to form a library of sequences enriched for specificity and affinity to the target. This process is often repeated to further enrich the library, with each subsequent round of SELEX narrowing down the number of sequences to only the sequences in the library that possess the highest level of specificity and affinity to the target. This reiterative process is a key defining aspect of SELEX. An outline of SELEX is presented in **Figure** **3**.

### 2.1 Engineering the Aptamer Discovery Process

Multiple variations of the SELEX process have been developed and are used in contemporary research, such as capillary electrophoresis (CE)‐SELEX,^[^ ^29^ ^]^ capture‐SELEX,^[^ ^30^ ^]^ cell‐SELEX,^[^ ^31^ ^]^ microphysiological system (MPS)‐based SELEX,^[^ ^32^ ^]^ magnetic bead‐SELEX,^[^ ^33^ ^]^ microfluidic (M)‐SELEX,^[^ ^34^ ^]^ and surface plasmon resonance (SPR)‐based SELEX.^[^ ^35^ ^]^ Most of these SELEX variations ultimately rely on the same general steps and reiterative selection process of SELEX as described above. Groups have also explored non‐SELEX methods of discovering aptamers,^[^ ^36^, ^37^, ^38^, ^39^, ^40^ ^]^ though these methods have not gained widespread adoption. Berezovski and colleagues described a method in which the reiterative amplification steps were omitted, but reiterative partitioning was still required and performed by a modified capillary electrophoresis technique in order to find aptamers against the h‐Ras protein.^[^ ^36^, ^37^ ^]^ Working on a larger target, *Escherichia coli*, Kim and colleagues also described a method in which there was no iterative amplification, but exploited the larger mass of their target to be able to use reiterative partitioning by centrifugation.^[^ ^40^ ^]^ Nitsche and colleagues described a non‐iterative, one‐step method to find aptamers against vaccinia virus, by using an affinity column to partition the virus‐binding oligonucleotides from the non‐binding oligonucleotides.^[^ ^38^ ^]^ Yoshikawa and colleagues developed a new aptamer discovery process based on sequencing, able to discover aptamers with high affinity and specificity for their target in a single selection step and without a counterselection step, observing essentially no off‐target binding, with the ability to differentiate between molecules that differ by only a single hydroxyl group.^[^ ^41^ ^]^ Recognizing that in some applications, the highest binding affinity aptamer may not be the most desired, Chang and colleagues developed a Pro‐SELEX methodology in which aptamers with customizable binding affinities are discovered in a single round of selection.^[^ ^42^ ^]^ In a notable non‐SELEX example, Yang and colleagues reported a functional‐group‐guided aptamer discovery strategy for small molecules, where systematic chemical‐moiety analysis and library partitioning enabled selective isolation of binders even for challenging low‐molecular‐weight targets.^[^ ^43^ ^]^ While SELEX‐based methods remain the gold standard for aptamer discovery, the development and adoption of non‐SELEX methods that eliminate repetitive steps are strongly needed to increase the rate of aptamer discovery. Although the authors of some studies which reduce their aptamer selection to only a single step still refer to their methodology as a variation of SELEX, we contend that a fundamental aspect of SELEX is the reiterative selection process required to enrich the starting pool to isolate a few or even one candidate aptamer, and thus methods based on a single selection step should be considered as non‐SELEX.

The choice of SELEX variation is often guided by the nature of the intended application and the biophysical characteristics of the target. For instance, when developing aptamers for cellular targets, methods such as cell‐SELEX are advantageous because they accommodate complex and heterogeneous surfaces, enabling the selection of aptamers that recognize and distinguish between membrane‐bound epitopes on the cell surface.^[^ ^31^ ^]^ Like cells, selecting aptamers for small‐molecule targets also presents unique challenges, due to their low molecular weight, limited surface area, and lack of multiple binding epitopes, which reduce opportunities for strong or multivalent interactions. In these cases, SELEX protocols often require modifications in partitioning or immobilization strategies to ensure efficient discrimination between bound and unbound species. For example, capture‐SELEX favors the evolution of structure‐switching aptamers that undergo a conformational change upon target binding, which can be harnessed to produce a measurable signal change in biosensors.^[^ ^44^, ^45^ ^]^ This is particularly important for small‐molecule detection, where mass‐based transduction methods, such as surface plasmon resonance (SPR) or quartz crystal microbalance (QCM), often lack sufficient sensitivity to detect the minute mass change associated with binding.^[^ ^46^, ^47^, ^48^ ^]^ In these scenarios, the binding‐induced folding or unfolding of the aptamer itself serves as an intrinsic amplification mechanism, converting molecular recognition into a fluorescence or electrochemical signal.^[^ ^49^, ^50^, ^51^ ^]^

The majority of aptamer discovery methods narrow down their starting library in their search for a select few final aptamers, at which point, the individual aptamer sequences can be characterized. The aptamers from these processes often have such high specificity for their target that a mutation on the target, producing a structural variance, may cause these ultra‐target‐specific aptamers to fail to recognize the mutated target, making these aptamers akin to the specific epitope recognition of monoclonal antibodies. Recognizing that there are situations where polyclonal antibodies may be more suitable than monoclonal antibodies, one group has developed polyclonal aptamer libraries, in which the polyclonal aptamer libraries are enriched for sequences with target‐specific affinity, but the number of individual unique sequences is only slightly narrowed down from the starting library. Developing a polyclonal library simplifies the aptamer discovery process by eliminating the need to sequence and analyze individual sequences to determine aptamer identity, and the larger sequence space has broader specificity, able to bind to more recognition sites on the target, decreasing the likelihood of failed recognition due to structural variance of the target. Their FluCell‐SELEX method has developed polyclonal aptamer libraries against a variety of bacteria^[^ ^52^, ^53^, ^54^ ^]^ and fungi,^[^ ^55^ ^]^ and they have also adapted the existing FluMag‐SELEX^[^ ^56^ ^]^ for developing a polyclonal aptamer library against a specific protein, retinol binding protein 4.^[^ ^57^ ^]^

### 2.2 Parameters of Aptamer Molecular Design

To tailor the aptamer, molecular engineering of the starting oligonucleotide library can be considered. Chemical modification of the oligonucleotide library serves two essential purposes: it increases the diversity of the library, making it more likely that a target‐binding aptamer will be found, and it can optimize the suitability of the aptamers for downstream applications (e.g., resistance to nucleases). Given the nature of aptamers as oligonucleotides, there are two obvious methods of modification: the identity of each nucleotide at a certain position (i.e., the sequence of nucleotides, or nucleobases), and the length of the molecule. While many oligonucleotide libraries for SELEX contain around 10^14^ – 10^15^ unique oligonucleotide sequences,^[^ ^10^, ^58^ ^]^ libraries may be larger, or have been observed to be as small as 10^6^ – 10^7^ unique sequences.^[^ ^59^, ^60^ ^]^ The typical length of each oligonucleotide is typically in the range of 20 – 100 nt.^[^ ^6^, ^61^ ^]^

**Figure 4:**
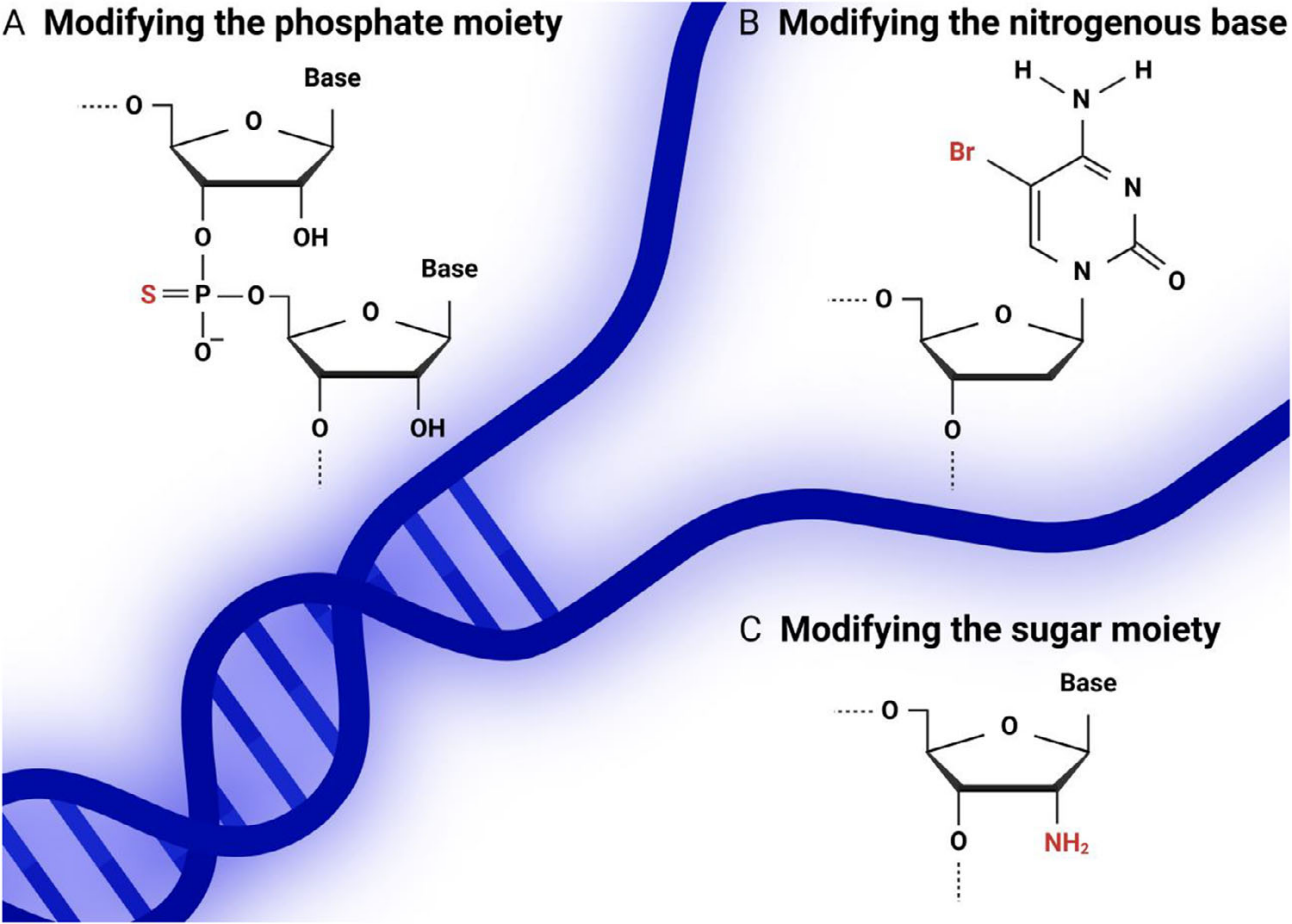
Examples of three types of nucleotide modifications applied to aptamers to increase their stability and functionality. A) Modification of the phosphate moiety, for example, by incorporation of a phosphorothioate linkage. B) Modification of the nitrogenous base, such as by incorporating bromine on carbon 5 of the pyrimidine ring. C) Modification of the sugar moiety, for example, by incorporating an amino group on the 2′ carbon.

While the initial discoveries and investigations of aptamers were on RNA aptamers, DNA aptamers were subsequently discovered and investigated soon after.^[^ ^62^, ^63^ ^]^ Between the two main classes of nucleic acids, there is no significant difference in binding affinity between DNA and RNA aptamers.^[^ ^64^ ^]^ Before screening, numerous chemical strategies have been developed to stabilize aptamers or modulate their binding characteristics, including sugar‐, base‐, and backbone‐level substitutions (e.g., 2′‐fluoro, 2′‐O‐methyl, LNA, or phosphorothioate modifications). The diversity of these chemistries reflects the wide range of structural and functional needs across different targets and assay conditions (**Figure** **4**). While naturally occurring oligonucleotides are in a right‐handed D‐DNA and D‐RNA conformation, left‐handed L‐DNA and L‐RNA can be synthetically produced and used for the synthesis of aptamers, with L‐RNA aptamers sometimes referred to as Spiegelmers. Xeno nucleic acids (XNA) are nucleic acid analogues synthesized with non‐natural phosphate backbones, sugars, or nucleotide bases. One noteworthy type of XNA is the locked nucleic acid (LNA) or bridged nucleic acid (BNA), both of which refer to modified RNA in which an extra carbon atom acts as a bridge between the 2' oxygen and 4' carbon, locking the conformation of the nucleic acid molecule. Literature searches describe the investigation of aptamers composed of a variety of different chemically modified nucleic acids, a selection of which are noted in **Table** **1**.

**Table 1:** Selected nucleic acid modifications for aptamer candidates.

Before or After Screening	Modification	Refs.
Before Screening	2′‐amino ribose	[77]
	2'‐fluoro‐arabinonucleic acid (FANA)	[73]
	2′‐fluro ribose	[78]
	2′‐O‐methyl ribose	[79]
	5‐bromo‐2′‐deoxyuridine	[80]
	Arabinonucleic acid (ANA)	[73]
	Cyclohexenyl nucleic acid (CeNA)	[73]
	Hexitol nucleic acid (HNA)	[73]
	Left‐handed DNA (L‐DNA)	[81]
	Left‐handed RNA (L‐RNA)	[82, 83]
	Locked nucleic acid/Bridged nucleic acid (LNA/BNA)	[84]
	Phosphorothioate	[85]
	Threose nucleic acid (TNA)	[86]
Before and After Screening	Polyethylene glycol (PEG)	[85, 87]
After Screening	^18^F‐fluoride	[88]
	Cells	[89]
	Enzymes	[90]
	Gold nanoparticles	[91]
	Graphene oxide‐coated nanoparticles	[92]
	Quantum dots	[93]

To further increase the diversity of nucleic acid libraries, unnatural base pairs (UBPs) expanding the diversity of the genetic alphabet beyond the canonical nucleotide bases (adenine [A], cytosine [C], guanine [G], thymine [T]), have been developed. Examples of such UBPs include the Ds‐Px pair,^[^ ^65^, ^66^, ^67^ ^]^ d5SICS–dNaM pair,^[^ ^68^ ^]^ AEGIS bases,^[^ ^69^, ^70^ ^]^ and the dMTMO‐dTPT3 and dPTMO‐dTPT3 pairs.^[^ ^71^ ^]^ Because modifications to the nucleic acid backbone, sugar moiety, and nucleotide bases may impact its structure, these chemical modifications are generally introduced before the nucleic acid library is screened against the desired target for binding. A challenge with using modified nucleic acids for the synthesis of aptamers is that they may not be able to be synthesized or replicated by conventional nucleic acid polymerases, although mutant or evolved polymerases have been developed, which overcome this limitation for certain modifications.^[^ ^72^, ^73^, ^74^, ^75^ ^]^ While solid‐phase synthesis allows incorporation of a broad spectrum of chemical modifications into aptamer sequences, its implementation is often constrained by the need for dedicated synthesizers and proprietary reagents. These factors make it less accessible to laboratories without advanced oligonucleotide synthesis facilities. Consequently, most aptamers are still generated using enzymatic methods with standard polymerases, which are simpler and more economical, but limited in the types of modifications they can accommodate.^[^ ^76^ ^]^

PEGylation is also a common chemical modification strategy for aptamers. Although there was at least one study that utilized a PEGylated nucleic acid library before screening against the desired target for binding affinity,^[^ ^87^ ^]^ the majority of reported studies PEGylated aptamers after screening.^[^ ^85^, ^94^ ^]^ This is because PEGylation is typically used in therapeutics to impart desirable in vivo properties, and not as a method to increase starting library diversity, which explains why PEGylation often is done on an already existing aptamer with known target‐binding action.^[^ ^95^ ^]^

Hybrid, chimeric, or conjugate aptamers may also be considered chemical modifications, as the aptamers are chemically coupled to another molecule, typically to enhance their function for the downstream application. It should be noted that in most instances of coupled aptamers, the aptamers have already been screened for affinity to the target, and so the coupling does not increase the diversity of the oligonucleotide library. Given the focus on engineering aptamers for diagnostics and sensing applications, most instances of coupled aptamers in this area of application are to enable either fluorometric or colorimetric visualization in the presence of the target to be detected. For instance, recent literature points to studies on the coupling of aptamers to gold nanoparticles,^[^ ^91^ ^]^ graphene oxide‐coated nanoparticles,^[^ ^92^ ^]^ and quantum dots^[^ ^93^ ^]^ or even labeling of the aptamer with radioisotopes such as ^18^F‐fluoride,^[^ ^88^ ^]^ all to permit visualization for diagnostics and sensing. Additionally, coupling individual aptamer sequences to larger carriers, such as beads or nanoparticles, can facilitate screening and analysis, as each carrier presents multiple copies of the same aptamer on its surface. This multivalent display enhances detectability compared to standard SELEX, where sequences of interest may be present at very low abundance during the early selection rounds. A few groups have already reported on one‐bead‐one‐compound synthesis of aptamer libraries, in which one aptamer sequence appears on one bead.^[^ ^96^, ^97^, ^98^, ^99^ ^]^ Coupling is not only limited to abiotic partners, as recent work has also produced cells^[^ ^89^ ^]^ and enzymes^[^ ^90^ ^]^ coupled to aptamers to permit diagnostics and sensing.

### 2.3 Mapping Functional Regions to Guide Aptamer Optimization

**Figure 5:**
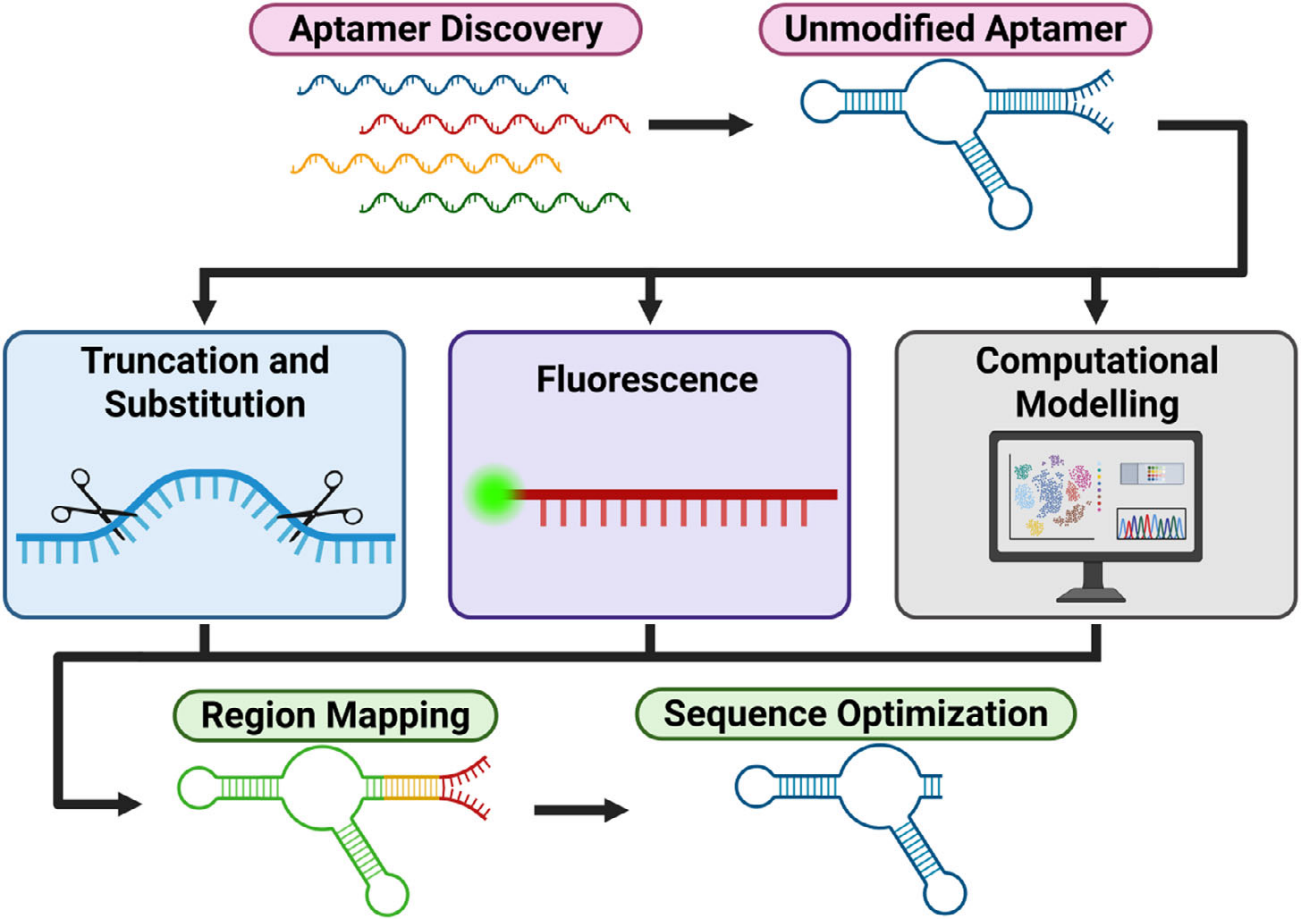
Complementary molecular engineering strategies that are applied to aptamers. Truncation and substitution will modify either/both the sequence length and base composition. Fluorescent‐based methods assess binding activity and target interaction. Computational modeling can predict aptamer structure and target‐binding ability. These methods can map functional regions in aptamers and can be used to optimize the aptamer sequence, producing shorter, higher‐affinity aptamers.

Aside from the analytical techniques used to characterize aptamers, molecular engineering strategies for aptamers (applied to aptamers with already confirmed target affinity) are often used to figure out two complementary aspects of aptamers: their essential and non‐essential regions. Knowing the essential regions allows for target‐binding optimization, providing information on which regions are significant for target‐specific binding, which regions should be preserved, or perhaps which regions can be further investigated to increase (or decrease) the binding affinity of the aptamer to the target. Knowing the non‐essential regions allows for sequence optimization, eliminating unnecessary nucleotides to get a more compact sequence, which can simplify the aptamer, reducing size, synthesis time, production cost, and may even increase binding affinity. Currently, the most common strategies used to map aptamer binding regions and optimize sequences include truncation and substitution, fluorescence‐based methods, and computational methods (**Figure** **5**), which will be introduced in Sections 3.3.1. through Section 3.3.3.

#### 2.3.1 Truncation and Substitution

Mapping and optimization of aptamers by truncation is a systematic approach to identify and refine the functional regions of aptamers. In aptamers, not all regions of the nucleic acid sequence contribute equally to their binding affinity and specificity. Mapping and optimization by truncation help pinpoint the essential regions responsible for their functionality while discarding non‐essential sequences. Truncation must preserve the secondary and tertiary structures essential for target binding.

In one interesting study, Dhiman and colleagues studied a snake venom binding aptamer known to bind to the α‐Toxin of *Bungarus multicinctus*, and truncated the aptamer to see if it was able to bind to the venom of *Bungarus caeruleus*.^[^ ^100^ ^]^ Their study noted that a truncated version of the aptamer was able to maintain target‐binding affinity to the original α‐Toxin of *Bungarus multicinctus*, while also being reactive to the cross‐species venom of *Bungarus caeruleus*, thus demonstrating the ability of a shorter aptamer that doesn't compromise affinity. Aljohani and colleagues studied a 60 nt long aptamer for the anti‐coagulant dabigatran etexilate and produced three differentially truncated sequences from the original 60 nt long aptamer.^[^ ^101^ ^]^ One of those three truncated sequences, a 38 nt long aptamer, showed a 47‐fold higher *K*_D_ than the original 60 nt aptamer. This demonstrated that a truncated aptamer can possess even superior binding affinity than an original, unoptimized aptamer. The addition of in silico methods can also be used to guide aptamer truncation, as illustrated by the work of Ma and colleagues studying aptamers against T‐2 mycotoxin and Aflatoxin B1, two dangerous food contaminants.^[^ ^102^ ^]^ Using molecular docking simulations, the authors predicted the binding domains in the aptamer‐target complex, and removed bases not relevant in the binding process, truncating the 80‐nt‐long T‐2 mycotoxin‐binding aptamer to 40 nt and truncating the 50‐nt‐long Aflatoxin B1‐binding aptamer to 32 bases. Additionally, their truncated 40 nt T‐2 mycotoxin‐binding aptamer was shown to have improved binding affinity compared to the original, non‐truncated aptamer.

In cases where an aptamer of a certain length is desired, it may be more feasible to map and optimize an aptamer by substitution. While mapping by substitution will provide limited insight into the non‐essential regions, it will provide insight into the essential regions by disruption (or enhancement) of secondary and tertiary structures, which may be important for binding. Manuel and colleagues demonstrated this effect in their study, which replaced guanosine with inosine in a cocaine‐binding aptamer. After the substitution, they generated aptamers with binding affinities ranging from 230 nM to 80 µM, including one generated aptamer with a single inosine substitution, which had a 63‐fold stronger binding affinity than the parent aptamer.^[^ ^103^ ^]^

#### 2.3.2 Fluorescence

Mapping the binding regions of aptamers is a crucial step in understanding how these molecules interact with their targets. Fluorescence‐based methods offer a sensitive, versatile, and non‐destructive approach to identifying the specific regions of aptamers that are directly involved in binding. These methods rely on the principle that fluorescence signals can change when an aptamer interacts with its target, providing valuable insights into binding dynamics and structural changes. Fluorophore placement must not interfere with aptamer folding or binding to ensure that 1) the aptamer's confirmation is not distorted and prevents the binding of the aptamer to the target, and 2) fluorescence changes result from aptamer‐target binding and not from non‐specific interaction of the fluorophore with the target.

Several studies have already shown that upon binding with its target, aptamers in the presence of an intercalating dye, such as SYBR Green I, may show a decrease in fluorescence as they lose their self‐annealed double‐stranded DNA regions and adopt the conformation required to bind to their target.^[^ ^104^, ^105^ ^]^ The target‐binding conformation may possess more single‐stranded DNA and lose its complex with the intercalating dye. While this approach supports that an aptamer binds to a target and changes conformation, it does not provide resolution on the specific regions or nucleotides that are responsible for binding. Rather, to map aptamer binding regions at greater resolution, individual nucleotides can be labeled. Photophysical properties (i.e., absorption and/or emission wavelength, emission intensity, fluorescence lifetime, and anisotropy) of fluorescent probes are altered by the local environment of the probe, and so conformational changes during target binding will provide a change in a photophysical property. For example, Zhang and colleagues were able to map aptamer‐protein interactions at the single‐nucleotide level by fluorescently labelling specific nucleotides with tetramethylrhodamine and evaluating the change in anisotropy between the aptamer‐protein complex and the unbound aptamer.^[^ ^106^ ^]^ Their work supported that the change in anisotropy was mostly due to the change in distance between specific nucleotides and the target protein, and that changes in anisotropy due to the structural changes that lead to alternative aptamer conformations were negligible.

#### 2.3.3 Computational Prediction and Modeling

Computational modeling has become a powerful tool for mapping the binding regions of aptamers. These models use advanced algorithms, molecular dynamics simulations, and structural data to predict how an aptamer interacts with its target, helping identify key regions responsible for binding and function. This approach complements experimental methods and can save time and resources by narrowing down regions for further study. Computational predictions and modeling depend largely on the accuracy of the input structures and parameters, and errors or excessive simplification of either the data or modeling algorithm can lead to misleading results.^[^ ^107^ ^]^ Therefore, all computational predictions must be validated experimentally to ensure their reliability.^[^ ^108^ ^]^ In addition, large or highly flexible targets may pose particular challenges for accurate modeling,^[^ ^109^ ^]^ and high‐resolution simulations, while more precise, require significant computational power and time.^[^ ^110^ ^]^ Nevertheless, computational modeling is especially useful when both the aptamer and its target are already well‐characterized, as this allows for more confident and informative simulations.

Typically, the prediction of single‐stranded oligonucleotide secondary (2D) structure is based on nearest neighbor thermodynamic models,^[^ ^111^ ^]^ which were typically determined by optical melting experiments.^[^ ^112^ ^]^ Some currently popular tools for predicting single‐stranded oligonucleotide (2D) secondary structure are mfold/UNAfold,^[^ ^113^, ^114^ ^]^ RNAfold,^[^ ^115^, ^116^ ^]^ and RNAstructure.^[^ ^117^, ^118^ ^]^ Although these tools haven't been designed specifically for aptamers, they are still generally applied to predict aptamer secondary structure. There is continuing work on tools for predicting tertiary (3D) structure, with several groups reporting on tools to predict oligonucleotide/aptamer tertiary structure alone,^[^ ^119^, ^120^, ^121^, ^122^, ^123^ ^]^ and complexed to a target,^[^ ^124^, ^125^, ^126^, ^127^ ^]^ with some of those more recent techniques leveraging the capabilities of AI.

The most recently exciting tool for biomolecular prediction, the AI program AlphaFold, has been noted to be the most accurate predictor of protein tertiary structure,^[^ ^128^ ^]^ and recent updates to AlphaFold 3 have expanded its capabilities to predict the structure of protein complexes with DNA, RNA, post‐translational modifications, and selected ligands and ions.^[^ ^129^ ^]^ However, its protein structure prediction is still not perfect, and an analysis of its capabilities shows that it does not flawlessly capture precise details of selected RNA‐ligand interactions, and in some cases, performs worse than other existing modeling tools.^[^ ^130^ ^]^ The same caveat likely also applies to aptamers, as aptamer‐target interactions often involve DNA or RNA oligonucleotides binding to proteins, small molecules, ions, or cells, and these binding modes can involve large conformational changes, induced fit, or flexible structural rearrangements.^[^ ^61^, ^131^ ^]^ AlphaFold 3 models are trained primarily on protein sequences, not on nucleic acid binders with such dynamic behavior. A recent study evaluating the modeling of DNA and RNA aptamers using AlphaFold 3 reported that, although the tool could successfully model certain aptamer structures, including motifs such as G‐quadruplexes, it was less reliable in predicting other noncanonical secondary structures, including pseudoknots and long‐range nucleobase interactions, indicating its current limitations in nucleic acid folding predictions.^[^ ^132^ ^]^ Nonetheless, the current computational tools have matured to the point of being able to generate new candidate aptamers for further investigation.^[^ ^133^ ^]^

## 3 Analytical Techniques for Screening and Characterization

**Figure 6:**
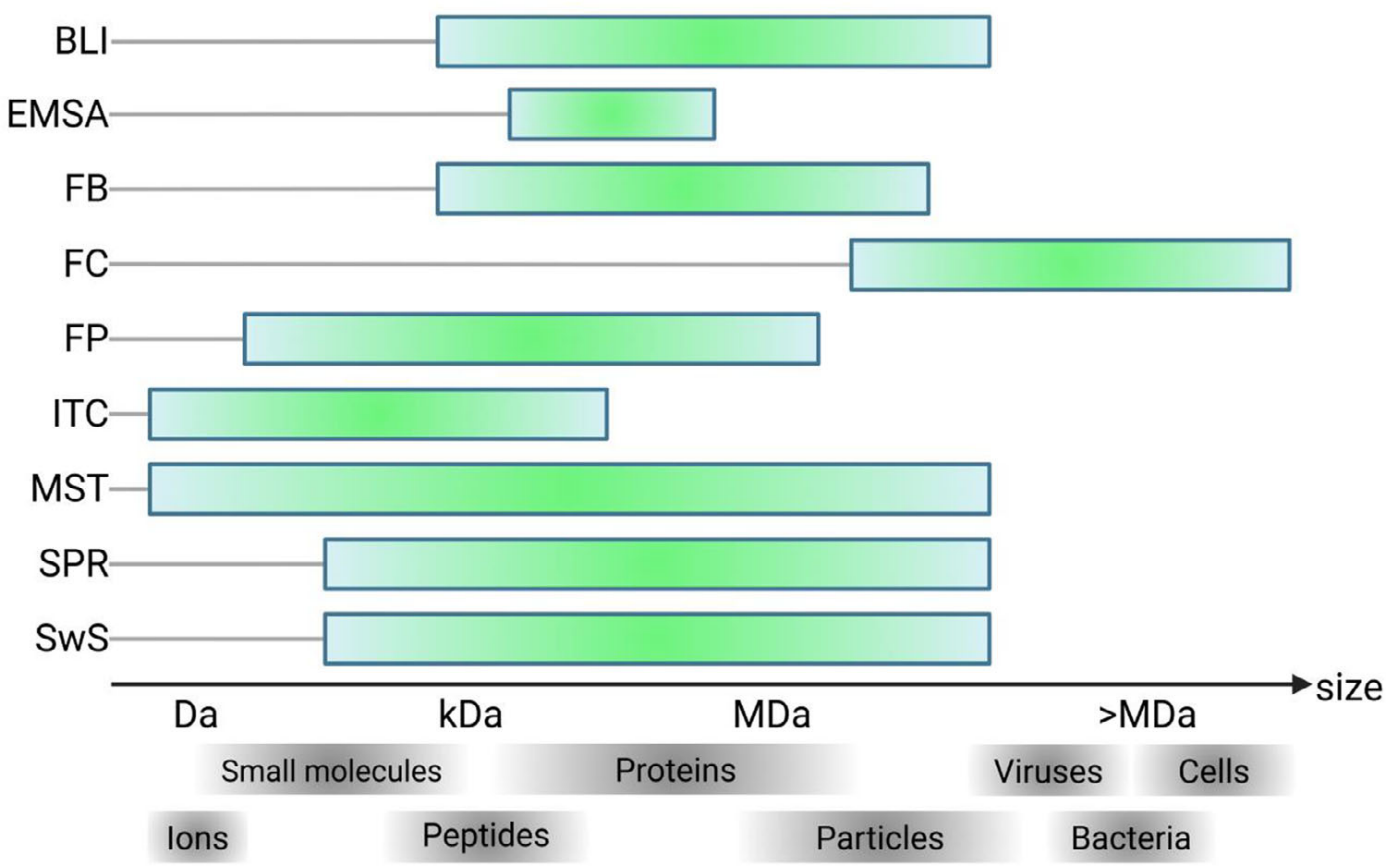
Application range of selected biophysical methods for studying interactions between aptamers and different target classes. The size of the aptamer target on the x‐axis increases from left to right (from ion to cells; from Da to >MDa). The application range of each biophysical method is sketched by a color gradient. Bright green shades indicate optimal application ranges. Redrawn from ^[^ ^135^ ^]^. Abbreviations: BLI, bio‐layer interferometry; EMSA, electrophoretic mobility shift assay; FB, filter‐binding assay; FC, flow cytometry; FP, fluorescence polarization; ITC, isothermal titration calorimetry; MST, microscale thermophoresis; SPR, surface plasmon resonance; SwS, switchSENSE.

Biophysical methods play a central role in drug discovery^[^ ^134^ ^]^ as they allow researchers to elucidate and characterize interactions between two components within a controlled environment, independent of the complexities inherent in cellular or animal models. They add a further benefit in the context of aptamer development. Many frequently used techniques in aptamer development are for historical and practical reasons based upon DNA‐readout, either by direct detection or by amplification. These approaches require a complementary strand for hybridization, which reduces the generalizability of a method. Several biophysical techniques commonly used in the development of small molecules or therapeutic proteins are also used for aptamers and are not limited by these detection methods. While there are many such methods that can assess biophysical interactions, they may differ in the exact information provided, or may be constrained in some aspects, such as only being able to assess binding partners within a certain mass (**Figure** **6**). The following section focuses on some frequently used techniques and their specific impact in assessing the binding between an aptamer and its target.

### 3.1 Differential Scanning Fluorimetry

Differential scanning fluorimetry (DSF) generally makes use of a change in a protein property whilst unfolding, running a temperature gradient. The readout can be based either on the binding of a fluorescent dye, as in the thermal shift assay (TSA), or on modulations of the protein's intrinsic fluorescence properties, as detected by nanoDSF, during unfolding of the protein. This allows them to identify the proteins' melting or infliction point, i.e., the temperature at which the reporter changes signal. The binding of another partner unaffected by the reporter to the protein can now result in a change in the melting/infliction temperature. These shifts can either be positive or negative, depending on the overall impact of the binding event.

TSA has been the first DSF implementation, starting with SYPRO Orange, a dye that binds to hydrophobic patches that are solvent accessible upon protein unfolding.^[^ ^136^, ^137^ ^]^ The plate‐based format allows for easily scaled assays for high throughput screening (HTS) with inexpensive instrumentation (qPCR instrument) and simple automation capabilities. Consequently, TSA has seen respective applications in the pharmaceutical industry.^[^ ^138^, ^139^ ^]^

However, the use of fluorescent dyes comes with limitations to certain buffer conditions (e.g., detergents) and quenching effects. The later development of nanoDSF allowed for broadening the application by making use of the intrinsic tryptophan fluorescence of proteins. The capillary‐based technique reads the fluorescence at 330 and 350 nm. Changes in the environment of tryptophane residues upon unfolding result in a shift in the intensities at 330 to 350 nm, which subsequently allows for determining also the melting/infliction temperature.^[^ ^140^, ^141^, ^142^ ^]^ Though DSF techniques could also be used to determine affinities, they require relatively high protein concentrations (1–10 µM), which limits the dynamic range for affinity determination to weak affinities in the µM range.^[^ ^143^ ^]^

The advantages in scalability and inexpensive instrumentation have also resulted in some applications of especially TSA, with aptamers. Hu et al. have developed a TSA set‐up which makes use of SYBR, a DNA‐intercalating agent that only binds to aptamers once they are folded.^[^ ^144^ ^]^ Unlike the conventional approach, where the dye binds to the protein, they have put their focus instead on the melting temperature of the aptamer. Hence, they have eventually compared the melting temperatures of the aptamers with and without protein supplementation. This setup comes with two major advantages over the common usage of TSA in small molecule‐protein interactions. First, the intercalating dyes are less restrictive to buffer composition, unlike SYPRO Orange. Second, the specificity of the dye for the aptamer allows work with unpurified proteins such as patient blood samples or lysates. The autofluorescent background can be subtracted, as the dye does not interfere with a complex protein matrix.

### 3.2 Microscale Thermophoresis

Microscale thermophoresis (MST) is a comparatively new technique having entered the market in the early 2010s. The theoretical background dates back to the 19th century, when the phenomenon of thermophoresis was first described. Thermophoresis refers to the diffusion of molecules along a thermal gradient, and its application to biological systems was demonstrated with DNA in 2010.^[^ ^145^, ^146^ ^]^ Molecules move in a thermogradient differently based upon changes in their effective charge, surface area, or hydration shell. Since binding of a molecule to another one can trigger these effects, e.g., by a conformational change, one will observe a difference in the movement between bound and unbound molecules. The technique uses fluorescence as a reporter for the movement, where commonly the protein is labelled and present at <10 nM concentrations. In order not to disturb the equilibrium critically, the applied thermogradient is maximally at 6K and initiated by an IR‐laser focused on a capillary. The capillary format of MST further has a beneficial effect on sample consumption: A single point measurement can be performed with 20 µl of the complex solution.

Due to the relativistic nature of MST, a titration series of the unlabeled binding partner is needed. This allows for differentiation of the diffusion behavior between the expected bound and unbound state and thus determines the affinity of the complex from a dose‐response plot. Highly sensitive fluorescence detectors in MST instruments allow for the detection of affinities down to the pM range.

MST requires labeling of one binding partner, which is often a protein. Common covalent labeling techniques via primary amines or thiols are available, but a set of labels for frequently used tags like His‐ and biotin‐tags can also be employed. The labeling can often be conceived as a disadvantage of the technique for protein‐protein or small molecule‐protein interactions; however, site‐specific labeling of aptamers is easily possible as part of the synthesis and mitigates many of the potentially undesired effects, like steric interference by the label.

The thermophoretic component of MST has frequently posed challenges in assay development. Though the technique can also work in very complex solutions, one often has to invest a lot of time in finding optimal buffer conditions. Since protein properties like surface charge are also affected by the buffer composition, focus has to be laid on validating the assay conditions properly. More recent developments from the original inventors offer substantial improvements in this regard. First‐generation dyes have been designed to be as inert as possible to changes in their environment. However, it was found that more environmentally sensitive dyes can make use of a phenomenon called Spectral Shift.^[^ ^147^ ^]^ When binding of a molecule to another molecule occurs in isothermal conditions (i.e., not under a thermogradient), subtle changes in the labeled binding partner can result in small but significant shifts in the emission spectra of a fluorescent probe. Therefore, one has only to read out the emission intensity at two slightly shifted wavelengths (e.g., 650 and 670 nm) and can deduce via a titration curve similarly.

The use of MST for aptamers was initially described by Baaske et al.^[^ ^148^ ^]^ Several works have proven the technique's capability in analyzing challenging interactions, including small molecules like ATP,^[^ ^149^ ^]^ drugs,^[^ ^150^ ^]^ protein‐glycoforms,^[^ ^151^ ^]^ or short peptides.^[^ ^152^ ^]^ The ability to determine affinities in complex solutions has been well documented by Rangel et al.^[^ ^153^ ^]^ They have developed threose nucleic acid aptamers of nM affinity against ochratoxin, a frequently found mycotoxin, and could detect the interaction also in 50% blood serum.

### 3.3 Surface Plasmon Resonance

Surface plasmon resonance (SPR) is a real‐time, label‐free technology employed to investigate the kinetics, affinities, and thermodynamics of two interacting partners. Unlike many other techniques, SPR is a surface‐based technology, i.e., one binding partner is immobilized on a sensor surface. The binding of an analyte injected into the flow cell results in a change in the refractive index, which is recorded. The binding event is thus recorded in real‐time, which gives access to the kinetic rate properties for association and dissociation of the complex. Other properties, such as complex half‐life, binding affinity (expressed as *K*_D_), or thermodynamic constant, are subsequently derived from single‐ or multi‐concentration series experiments.

A major aspect of every SPR is immobilization. The sensor surface is commonly gold, as it needs to be a noble metal. Blank gold surfaces are not favorable due to inhomogeneous surface properties; subsequently, the standard sensor has a self‐assembled monolayer formed by a thioalkane acid with a functional head group (often carboxylates). One interaction partner is then immobilized by covalent (chemical coupling reactions like amine coupling) or non‐covalent (tag‐based capture systems) approaches. It is typically more efficient to immobilize the target protein on the sensor surface and then use the aptamer developed against a specific protein as the injected analyte. However, if aptamers have been raised against molecule classes of low molecular weight (small molecules or peptides), immobilization of the non‐aptamer interaction partner comes with critical limitations, such as a lack of coupling‐reactive groups. Furthermore, immobilization of a small molecule can be problematic if the functional group required for aptamer binding becomes masked or inaccessible upon surface attachment. In these cases, an aptamer can be easily immobilized, given that the introduction of coupling reactive groups or tags like biotin can be easily incorporated during solid‐phase synthesis. Nevertheless, immobilization can restrict the structural flexibility of an aptamer, limiting its ability to undergo the conformational changes required for target binding. Chang et al. elegantly solved this problem by introducing a spacer sequence to the aptamer (e.g., a poly‐A spacer of 24 base pairs at 3’) and with the complementary sequence being immobilized on a sensor surface.^[^ ^154^ ^]^ With this approach, 11 aptamer‐small molecule interactions were characterized. The approach is also beneficial for assays of high throughput, as the linker can be easily regenerated without denaturing.

A traditional case of using SPR in aptamer development is reported by Schmitz et al.^[^ ^155^ ^]^ The research group developed aptamers raised against the surface protein of SARS‐CoV‐2 and validated their selected aptamers by SPR. Thereby, they immobilized biotinylated aptamers on the sensor surface and determined affinities at 25 and 37°C. Affinities in the range of 10–30 nM could be detected from the automated selection process.

Several research groups have made use of SPR to bypass the SELEX process by including SPR in the screening process. Due to its surface‐based nature, the SPR sensor can be used like a separation column in an HPLC, even on low‐throughput instruments. The basic principle is that high‐affinity binding aptamers bind to their target and thus have a longer retention time on the sensor. Consequently, they are eluted later or only after the injection of a solution. Single aptamer molecules are then fished out of the eluate and amplified by PCR. This also allows for the injection of a complete cocktail initially, as weak binders are automatically removed. The limitation of this approach is that affinities need to be in the nM to pM range with slow off‐rates. Weaker, but still potentially valuable hits, can thus go undetected. Kurt et al. have immobilized their target protein, the fibroblast growth receptor protein 3 K650E, on a self‐assembled monolayer sensor by amine coupling.^[^ ^156^ ^]^ Subsequently, they injected their complete library from several SELEX rounds and amplified hits from the eluate. A cross‐check for surface interaction and other negative controls could also be included similarly. Eventually, they found an aptamer of 28 nM.

A slightly modified attempt was reported by Jia et al.^[^ ^157^ ^]^ A critical aspect of using SPR as a surface‐retention module is the signal amplitude, i.e., the instrument's capability of detecting binding. Assays against single proteins can be performed on a low‐throughput instrument with sufficiently high affinity, but if additional controls or selectivity‐relevant proteins are to be included, a multiplexing SPR is required. The research group decided to build its own imaging SPR. Since the SPR signal can be increased by adding mass to an analyte, they increased responses by immobilizing the aptamers on silver nanoparticles. They have observed over several SELEX rounds the improvement in binders to their target protein, Lactoferrin, and unspecific binding behavior to several negative controls. Nine aptamers of nM‐affinity could be identified after six SELEX rounds.

### 3.4 Additional Biophysical Methods

An important step in facilitating the current aptamer development process lies not only in faster screening methods, but also in additional control mechanisms. Amano et al. have developed a nuclear magnetic resonance (NMR) methodology based on 1D‐imino proton NMR analysis, which aids in supervising the enrichment of a library during the SELEX process.^[^ ^158^ ^]^ NMR has the advantage that it's non‐destructive and gives orthogonal insight into the formation of secondary structures.

Another attractive technique that has emerged in recent years is native electrospray mass spectrometry. Gülbakan et al. showed for three different aptamer model systems how native ESI‐MS can easily gain access to higher‐order structure information, stoichiometry, and binding modes.^[^ ^159^ ^]^ Such information can be critical in identifying potential candidates based on more than affinities in a comparably simple and feasible manner compared to X‐ray or NMR analysis. Similarly, Daems et al. have developed an ion‐mobility mass spectrometry workflow and tested it with cocaine‐binding aptamers.^[^ ^160^ ^]^ Higher‐order structure information, insight into binding modes, and a relative ranking were gained. Since relative ranking is also possible by both native ESI‐ and IM‐MS, mass spectrometry as a technique can also be exploited for screening. However, MS has not yet been widely employed as a screening technique for aptamers despite its easy integration into automated workflows.

Quartz crystal microbalance with dissipation monitoring (QCM‐D) has also become an increasingly powerful analytical tool for characterizing aptamer–target interactions. This technique enables real‐time monitoring of changes in mass and viscoelastic properties at the sensor surface as aptamers bind to their targets, providing quantitative information on binding affinity, kinetics, and conformational dynamics. For instance, in the study by Osypova and colleagues, QCM‐D was employed to reveal folding‐induced frequency and dissipation shifts associated with aptamer–analyte binding, demonstrating its capability to distinguish structural transitions linked to recognition events.^[^ ^161^ ^]^ Likewise, the work reported by Nakatsuka and colleagues highlighted how surface‐sensitive approaches such as QCM‐D can capture conformational rearrangements during small‐molecule binding, offering complementary insight into aptamer functionality beyond simple affinity measurements.^[^ ^162^ ^]^ Together, these studies underscore the value of QCM‐D as a label‐free and highly sensitive method for elucidating the mechanistic aspects of aptamer recognition that are essential for biosensor optimization and rational aptamer engineering. These techniques, in addition to other selected analytical techniques, are noted in **Table** **2**.

**Table 2:** Selected screening and characterization methods for analyzing aptamer‐target interactions.

Analytical Technique	Principle	Information Obtained	Refs.
Biolayer interferometry (BLI)	Change in reflection patterns upon interaction of a binding partner with the other partner immobilized on a sensor tip	Binding affinity, Binding kinetics	[168]
Differential scanning fluorimetry (DSF)	Change in the fluorescence intensity of dye or intrinsic fluorescence due to the unfolding process of the protein upon interaction with the binding partner	Melting/inflection temperature	[138, 141]
Circular dichroism (CD) spectroscopy	Enantiomeric substances absorb circularly polarized light differently	Secondary structure elucidation	[169]
Electrophoretic mobility shift assay (EMSA)	Change in molecular/complex size changes the electrophoretic mobility	Binding affinity	[170]
Flow cytometry (FC)	Change in the fluorescence intensity	Binding affinity	[171]
Fluorescence polarization (FP) assay	Change in rotational speed and thus fluorescence polarization due to interaction with the binding partner	Binding affinity, Competition constants	[172]
Gel electrophoresis, including method variations except EMSA	Charged species migrate toward the electrode of opposite charge, and molecular size changes the electrophoretic mobility	Binding affinity, Molecular size/sequence length	[173]
Isothermal titration calorimetry (ITC)	Change in heat between the measurement and reference chamber upon titration of binding partner to other partner in solution at isothermal conditions	Binding affinity, Stoichiometry, Thermodynamics	[174]
Microscale thermophoresis (MST)	Change in chemical microenvironment of fluorescent probe on one binding partner indicates interaction with other binding partner, either in isothermal (Spectral Shift) or thermogradient (Microscale Thermophoresis)	Binding affinity, Competition constants, Thermodynamics	[175]
Nuclear magnetic resonance (NMR) spectroscopy	Detection of emitted electromagnetic waves of certain atomic nuclei upon perturbation by a weak magnetic oscillating field and depending on the chemical environment of said nuclei	Structure determination, Binding affinity, Competition constants	[176]
Quartz crystal microbalance with dissipation monitoring (QCM‐D)	Measures the change in resonant frequency and energy dissipation of a quartz crystal sensor upon adsorption or desorption of molecules, reflecting mass and viscoelastic properties of bound layers in real time.	Binding affinity, Binding kinetics, Surface mass change, Film viscoelasticity	[177, 178]
Surface plasmon resonance (SPR) imaging/microscopy (SPRi/SPRM)	Changes in refractive index indicate interaction between binding partners	Binding affinity, Binding kinetics, Thermodynamics, Competition constants, Binding mode	[168]
switchSENSE (SwS)	Change in the local environment of dye (static mode) or in the movement of DNA‐level in oscillating electric field (dynamic mode)	Binding affinity, Binding kinetics, Thermodynamics, Competition constants, Binding mode/Conformational changes	[179]

Beyond these experimental approaches, in silico molecular dynamics (MD) simulations provide atomistic, time‐resolved insight into aptamer‐target recognition that complements biosensor readouts. MD simulates the physical movements of the atoms and molecules over a fixed time, providing insight into the dynamics of the interaction between two partners, which can be between an aptamer and its target. While we previously discussed in silico structure prediction in Section 3.3.3., we alluded to the fact that some of those methods can predict potential 2D and 3D aptamer structures (in isolation) based on their nucleobase sequence, with some of the newer AI‐based approaches proposing novel aptamer candidates that take into consideration the desired target's conformation. These previously discussed predictors can give rudimentary insight into the potential structure of an aptamer, guide the design of an aptamer library, or screen potential aptamer targets for further study. What we think distinguishes MD simulations from these other techniques is that upon obtaining the structure of an aptamer (and its target), MD simulations can characterize the interaction. This structural data can come from analytical instrumentation, which would provide the actual, real‐world structure, or be predicted/modeled from in silico methods. Online databases or repositories may contain structures for some of these previously characterized molecules (including biological macromolecules).^[^ ^163^, ^164^, ^165^ ^]^ Studies have used MD simulations not only to characterize the aptamer‐target interaction, but also to gain an understanding of their aptamer‐functionalized sensor.^[^ ^166^, ^167^ ^]^

## 4 Aptasensors

**Figure 7:**
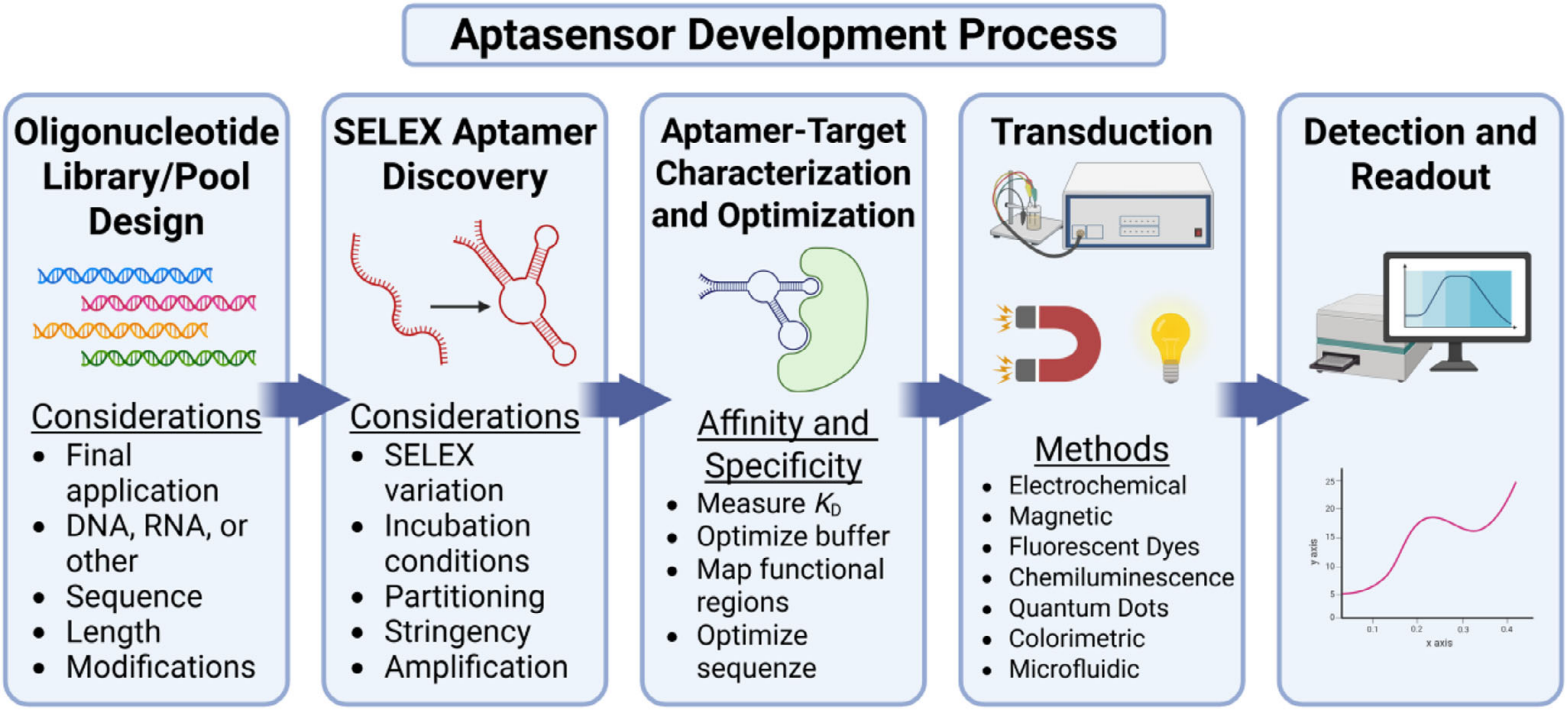
Schematic representation of the general aptasensor development process. The process begins with an oligonucleotide library or pool. When selecting aptamers, the final application must be considered (e.g., structure‐switch selection is a common method for ensuring aptamers are suitable for aptasensors and are often critical when selecting for small‐molecule binding aptamers). Top aptamers must be characterized and optimized. The binding event must be transduced, which can be achieved using different signal conversion mechanisms. Finally, the detection system interprets and displays the signal, forming the functional aptasensor.

Biosensors have emerged as a vital technology in response to the critical need for sensitive and accurate detection of targets across a broad spectrum of applications, including environmental monitoring, food safety, law enforcement, and medical diagnostics. These devices utilize biological molecules as probes to achieve rapid and precise identification of specific analytes,^[^ ^180^, ^181^ ^]^ and the combination of aptamers with sensors is often referred to as aptasensors, and an overview of their development process is presented in **Figure** **7**.

Chemical forces such as hydrogen bonds,^[^ ^182^, ^183^, ^184^ ^]^ electrostatic interactions,^[^ ^183^, ^185^, ^186^ ^]^ hydrophobic effects,^[^ ^187^, ^188^ ^]^ π‐π stacking,^[^ ^189^, ^190^ ^]^ and van der Waals forces,^[^ ^191^, ^192^ ^]^ critically influence the dynamics of aptamer‐target interactions. These interactions facilitate the specific and strong binding of aptamers to their targets, crucial for the effective sensing of the target. Experimental assay designs like structure‐switching,^[^ ^193^, ^194^, ^195^, ^196^ ^]^ molecular proximity‐based designs,^[^ ^197^ ^]^ competitive displacement assays,^[^ ^198^, ^199^ ^]^ sandwich‐structure designs,^[^ ^198^, ^200^ ^]^ split aptamers,^[^ ^201^ ^]^ and nanoparticle conjugated aptameric biosensors^[^ ^202^, ^203^ ^]^ leverage these interactions to improve the detection and quantification of toxic analytes. These strategies enhance the sensitivity, specificity, and versatility of the aptasensors, making them powerful tools for monitoring toxic substances in various environments.^[^ ^204^, ^205^ ^]^

This capability plays a crucial role in detecting a diverse array of targets such as toxins, heavy metals, illicit drugs, and medical biomarkers like metabolites and neurotransmitters.^[^ ^23^, ^200^, ^206^, ^207^, ^208^ ^]^ The main advantage of aptasensors over other types of devices lies in their ability to integrate biological components, such as antibodies, protein receptors, biomimetic materials, and nucleic acids, with electronic systems to form reliable, often portable, tools for the quick and cost‐effective analysis of substances.^[^ ^209^ ^]^ Aptasensors translated to real‐world applications in medical diagnostics and non‐medical sensing will be discussed in Sections 6 and 7.

## 5 Medical Diagnostics Applications

**Figure 8:**
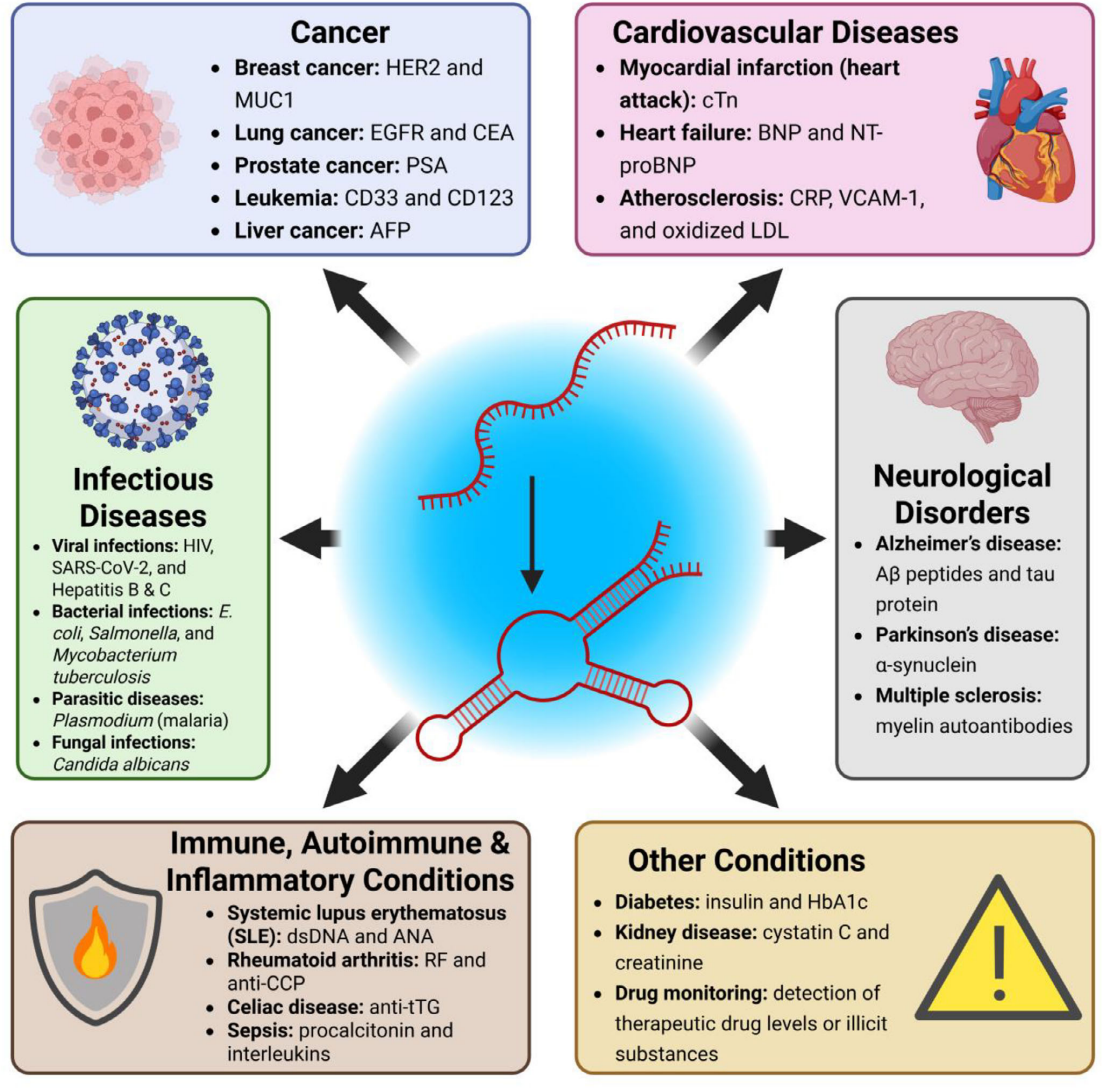
Overview of disease categories detectable using aptamer‐based diagnostics. Aptamers have been engineered to bind specific biomarkers associated with a wide range of conditions, including cancer, cardiovascular diseases, neurological disorders, infectious diseases, autoimmune and inflammatory diseases, and other conditions. Abbreviations: AFP, Alpha‐Fetoprotein; ANA, Antinuclear Antibody; anti‐CCP, Anti‐Cyclic Citrullinated Peptide antibody; anti‐tTG, Anti‐Tissue Transglutaminase antibody; BNP, B‐type Natriuretic Peptide; CEA, Carcinoembryonic Antigen; CRP, C‐reactive Protein; cTn, Cardiac Troponin; dsDNA, Double‐Stranded Deoxyribonucleic Acid; EGFR, Epidermal Growth Factor Receptor; HbA1c, Hemoglobin A1c; HER2, Human Epidermal Growth Factor Receptor 2; LDL, Low‐Density Lipoprotein; MUC1, Mucin 1; NT‐proBNP, N‐terminal prohormone of B‐type Natriuretic Peptide; PSA, Prostate‐Specific Antigen; RF, Rheumatoid Factor; SLE, Systemic Lupus Erythematosus; VCAM‐1, Vascular Cell Adhesion Molecule 1.

In recent years, the field of precision medicine has seen remarkable advancements, particularly in molecular and cellular diagnostic technologies. Integral to this progress are molecular recognition elements, essential for the development of rapid, reliable, and efficient diagnostic methods. Aptamers stand out as a pivotal tool in molecular analytics, offering manifold advantages ideally suited for diagnostic and biosensing applications. **Figure** **8** gives an overview of all of the conditions, and their exact biomarkers, against which aptamers have been developed for diagnostic purposes.

One of the key strengths of aptamers lies in their ability to adopt diverse 3D structures, facilitating target recognition with exceptional specificity and affinity.^[^ ^210^, ^211^, ^212^ ^]^ Unlike antibodies, aptamers' compact size allows for denser immobilization on sensor surfaces, potentially enhancing sensitivity.^[^ ^8^ ^]^ Moreover, aptamers exhibit superior stability and resilience against harsh environmental conditions, including temperature variations, pH shifts, humidity, and organic solvents.^[^ ^213^, ^214^, ^215^, ^216^ ^]^ Furthermore, aptamers are more amenable to chemical modifications, such as conjugation with fluorescent dyes, radionuclides, redox labels, and nanomaterials, offering greater versatility.^[^ ^217^ ^]^ Notably, the production of aptamers on a large scale incurs lower costs and exhibits reduced batch‐to‐batch variation compared to antibodies.^[^ ^218^, ^219^, ^220^ ^]^ Additionally, the programmable nature of aptamers enables customizable structural designs and engineering approaches, facilitating diverse strategies for signal amplification to improve target detection.^[^ ^221^ ^]^ More specifically, aptamers can incorporate diverse signal amplification components for direct or indirect enhancement, including nucleic acid primers and trigger sequences, functional labels, and nanomaterials. Direct amplification entails aptamer‐target binding triggering nucleic acid‐based reactions, while indirect methods rely on aptamer‐probe hybrids releasing trigger sequences for amplification. Common amplification strategies are summarized in **Table** **3**.

**Table 3:** Common strategies for signal amplification in aptamer‐based detection systems.

Amplification Strategy	Description	Comparative Features	Refs.
Rolling circle amplification (RCA)	Circular DNA templates continuously replicate upon target binding, initiating the RCA process, generating long repetitive sequences for enhanced signal.	Produces long, repetitive DNA strands for strong signal amplification; ideal for ultrasensitive detection but slower and enzyme‐dependent.	[222, 223, 224]
Hybridization chain reaction (HCR)	DNA hairpins self‐assemble upon target binding, initiating a cascade of hybridization events and formation of long DNA structures, amplifying the signal.	Enzyme‐free and highly programmable; suitable for isothermal amplification, but with slower reaction kinetics.	[225, 226]
DNAzyme‐based amplification	Catalytic DNAzymes cleave substrates in the presence of the target, generating a detectable signal.	Enables target‐triggered catalytic turnover and strong signal amplification; it works under mild conditions but is limited by substrate design.	[227]
Catalytic hairpin assembly (CHA)	DNA hairpins undergo structural changes upon target binding, initiating a self‐sustained amplification loop.	Enzyme‐free and fast amplification; low background noise, suitable for multiplexed biosensing.	[228, 229]
Nanoparticle‐based amplification	Aptamer‐nanoparticle complexes undergo conformational changes upon target binding, altering nanoparticle properties and enhancing signal through various mechanisms.	Provides strong signal amplification; allows visual or electrochemical readout, but may require complex nanomaterial synthesis.	[230]

Due to their numerous advantages, aptamers are being incorporated in biosensors and analytical devices for medical diagnosis,^[^ ^231^, ^232^ ^]^ bioimaging^[^ ^210^ ^]^ and biosensing,^[^ ^233^, ^234^ ^]^ targeted drug delivery,^[^ ^235^, ^236^ ^]^ food safety testing,^[^ ^237^, ^238^, ^239^ ^]^ and environmental monitoring.^[^ ^240^ ^]^ In this section, we review the latest developments in aptamer‐based clinical diagnosis, concentrating on applications in cancer, cardiovascular, and infectious diseases. The focus is on aptamer‐based technologies that have clinical translation potential based on their performance with clinically relevant samples and highlight research demonstrating a higher level of integration for point‐of‐care applications. In this section, we highlight and discuss the most significant examples that have shaped the progress in the area of clinical diagnosis.

### 5.1 Cancer

Cancer remains a major global health challenge, where early and accurate detection is critical for improving patient outcomes.^[^ ^241^ ^] [^ ^242^ ^]^ Aptamers are particularly well suited to oncology diagnostics because they can be engineered to recognize diverse cancer‐associated targets, ranging from soluble tumor antigens to circulating tumor cells and exosomes.

#### 5.1.1 Detection of Soluble Tumor Biomarkers

PSA represents one of the key biomarkers available for the early detection of prostate cancer.^[^ ^243^ ^]^ To date, a variety of aptamer‐based sensors for PSA detection in serum have been developed.^[^ ^244^, ^245^, ^246^, ^247^, ^248^, ^249^, ^250^, ^251^, ^252^, ^253^ ^]^ For instance, a label‐free colorimetric detection of PSA in serum using a cationic polymer and poly‐Adenine aptamer adsorbed on gold nanoparticles was demonstrated with a low limit of detection (LOD) of 20 pg mL^−1^.^[^ ^247^ ^]^ To increase the specificity of PSA screening for prostate cancer diagnosis, Diaz‐Fernandez et al. have deployed two aptamers for dual‐recognition of PSA using an impedimetric sensor consisting of nanostructured gold electrodes.^[^ ^250^ ^]^ The sensors are able to measure PSA in serum with a dynamic range between 0.26 and 62.5 ng mL^−1^. For highly heterogeneous diseases, such as cancer, sensitive and selective detection of multiple biomarkers is an effective diagnostic strategy in the early stages of disease. Xu et al. have developed a surface‐enhanced Raman scattering (SERS) encoded silver pyramid sensing system for multiplex biomarker detection using three different aptamers specific to PSA, thrombin, and MUC1.^[^ ^244^ ^]^ The linear range of the PSA sensor corresponded to 0.08 and 30 aM with a low limit of detection of 0.039 aM.

Measurement of human epidermal growth factor receptor‐2 (HER‐2/neu) levels in serum might play an essential role as a diagnostic/screening marker for breast cancer.^[^ ^254^, ^255^ ^]^ Different aptasensor approaches have been applied for the detection of HER‐2^257^ using electrochemical^[^ ^257^, ^258^, ^259^ ^]^ and optical detection schemes. For instance, an aptasensor for direct measurement of HER2 in undiluted serum using electrochemical impedance spectroscopy was developed using HER‐2‐specific aptamer immobilized on the surface of gold screen‐printed electrodes.^[^ ^260^ ^]^ The sensor provided a linear range of operation for concentrations ranging from 1 pg mL^−1^ to 100 ng mL^−1^ with an LOD of approximately 170 pg mL^−1^. A lower LOD of only 50 fg mL^−1^ was achieved using a HER‐2 aptasensor on a glassy carbon electrode, which combined the synergistic effects of gold nanoparticles, graphene oxide, and single‐walled carbon nanotubes, and was successfully validated using clinical samples from patients.^[^ ^261^ ^]^ Furthermore, an innovative electrochemical detection platform relying on aptamer‐analyte‐aptamer sandwich assay and DNA self‐assembly amplification was recently used for the detection of HER2 in human serum of breast cancer patients, providing an LOD of 0.047 pg mL^−1^ and demonstrated highly linear performance across its operating range.^[^ ^262^ ^]^ Toward point‐of‐care applications, an instrumentless colorimetric assay for detection of HER2 has also been developed using aptamer‐ gold‐nanoparticle conjugates, and its potential for point‐of‐care deployment was further demonstrated using lateral‐flow device.^[^ ^263^ ^]^

**Figure 9:**
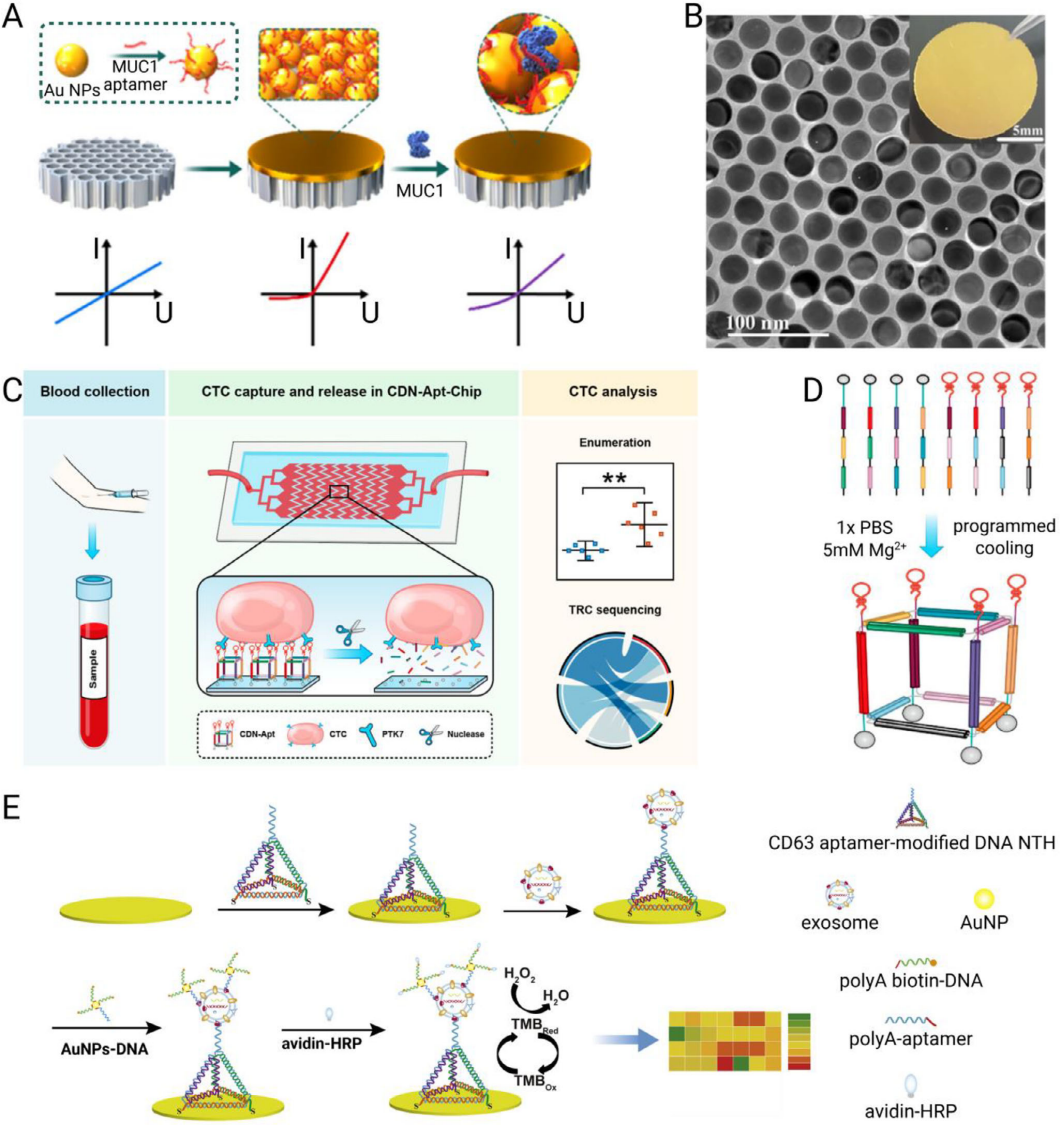
Aptamers applied to cancer diagnostics for detecting A,B) soluble tumor biomarkers, C,D) circulating tumor cells, and E) exosomes. (A) Current (I) and voltage (U) properties of bare anodized aluminum oxide (AAO), the modified gold nanofilm‐AAO (Au‐AAO) ion channel, and its binding with MUC1. B) TEM image of the aptamer‐modified monolayer gold nanofilm. The inset is a macroscopic photograph of the Au‐AAO ion channel; the diameter of the Au‐AAO nanofilm in the photograph is 12 mm. Adapted with permission.^[^ ^267^ ^]^ Copyright 2021, American Chemical Society. C) Schematic diagram depicting the workflow of Cubic DNA Nanostructure (CDN)‐Apt‐Chip for capture and release of CTCs for T‐cell Receptor sequencing. D) Schematic diagram showing the self‐assembly of CDN‐Apt. Adapted with permission.^[^ ^319^ ^]^ Copyright 2022, American Chemical Society. E) Schematic illustration of the aptasensor for exosomal protein profiling. Adapted with permission.^[^ ^320^ ^]^ Copyright 2020, Elsevier. Abbreviations: HRP, horseradish peroxidase; MUC1, mucin‐1; NP, nanoparticle; NTH, nanotetrahedron; PBS, phosphate buffered saline; TMB, 3,3′,5,5′‐tetramethylbenzidine.

Mucin 1 (or MUC1), a glycosylated protein present on the epithelial cell surface, has been shown to participate in the progression and metastasis of different cancers, including colon carcinoma, ovarian malignancy, renal tumors, breast cancer, and lung carcinoma.^[^ ^264^ ^]^ Consequently, a large number of aptamer‐based sensors have been developed in recent years for MUC1 detection.^[^ ^265^ ^]^ Among these, several sensors have been successfully deployed for MUC1 detection in serum with good sensitivity and specificity. For instance, Li et al. have integrated SERS probes with magnetic separation to develop a dual‐mode SERS‐colorimetric aptasensor providing the possibility for rapid instrumentless screening using colorimetry while preserving the capacity to achieve a low LOD of 0.1 U mL^−1^ with Raman signal detection.^[^ ^266^ ^]^ A novel aptamer‐gated ion channel constructed from nanoparticle self‐assembled gold nanofilm‐anodized aluminum oxide with an inter‐layer encoded aptamer was developed for sensitive electrochemical detection of MUC1 from clinically relevant samples with an LOD as low as 0.0364 fg mL^−1^ or 0.0025 aM (**Figure** **9**A,B).^[^ ^267^ ^]^ Zhou et al. have proposed a quantitative aptamer‐based MUC1 fluorescence detection scheme using aptamer‐tagged silver nanoclusters containing a recognition unit of MUC1 aptamer as the label‐free fluorescence probe.^[^ ^268^ ^]^ The sensor operated in a linear range for MUC1 concentrations between 0.1 to 100 nM, with a reported LOD of 0.05 nM. A different fluorescence detection strategy employing RCA and combining MUC‐1 aptamer and graphene oxide biointerface was successfully demonstrated in spiked human serum, urine, and saliva with a reported LOD of 10 pg mL^−1^.^[^ ^269^ ^]^ An electrochemical aptasensor utilizing a similar amplification scheme was demonstrated by Wen et al. with an LOD of 4 pM and a dynamic range of 10 pM to 1 µM.^[^ ^270^ ^]^ Toward the development of a point‐of‐care MUC1 analytical test, Ma et al. demonstrated a paper‐based microfluidic device with spiked serum samples that relies on an aptamer for specific target recognition and downstream HCR for electrochemiluminescent signal amplification, achieving an LOD of 8.33 pM.^[^ ^271^ ^]^

Osteopontin (OPN), a multifunctional protein present in blood and other bodily fluids, has been recognized as a potentially valuable biomarker for diagnosis and prognosis of cancer^[^ ^272^, ^273^, ^274^ ^]^ and nonmalignant diseases.^[^ ^275^, ^276^, ^277^ ^]^ A simple, label‐free electrochemical aptasensor for was developed by functionalizing screen‐printed gold electrodes with an OPN‐specific aptamer, and its performance was evaluated using square wave voltammetry measurements in serum to achieve an LOD of 1.4 nM.^[^ ^278^ ^]^ Toward the development of a point‐of‐care test strip, Pereira et al. introduced a paper‐based aptasensor for colorimetric OPN detection in spiked serum samples using Bradford reagent within 30 min, resulting in an LOD lower than 5 ng mL^−1^.^[^ ^279^ ^]^ A greater multiplexing capability in a lateral flow test strip was demonstrated by Li et al. for simultaneous detection of OPN and vascular endothelial growth factor (VEGF) using both colorimetric detection and SERS spectroscopy with LODs of 10 pg mL^−1^ and 0.8 pg mL^−1^, respectively.^[^ ^280^ ^]^ Using plasma samples of healthy controls and cervical cancer patients, the authors demonstrated that the 15‐min diagnostic test sensitivity and specificity were 92% and 94%, respectively, making it a promising tool for the early detection of cervical cancer in point‐of‐care settings.

Alpha‐fetoprotein (AFP) is one of the most widely used biomarkers for early diagnosis of individuals with liver cancer.^[^ ^281^, ^282^ ^]^ To date, various aptamer‐based AFP sensors have been demonstrated,^[^ ^283^ ^]^ with several assays showing potential for their use with complex matrices.^[^ ^284^, ^285^, ^286^, ^287^, ^288^, ^289^, ^290^, ^291^, ^292^, ^293^, ^294^, ^295^ ^]^ Toward sensitive and highly selective detection of AFP in clinical serum samples, Li et al. have developed an enzyme‐free fluorescence sensing scheme that relies on aptamer‐based AFP recognition followed by CHA signal amplification.^[^ ^285^ ^]^ The assay could be completed in 60 min and exhibited a low LOD of 0.033 ng mL^−1^ with a wide detection range from 0.1 ng mL^−1^ to 10 µg mL^−1^. An integrated aptasensing platform for voltammetric AFP detection on a graphene oxide nanosheet‐modified gold‐disk electrode embedded in a detection cell was demonstrated using Prussian blue nanoparticles for signal generation.^[^ ^290^ ^]^ Using the target RCA approach, the platform operated in a linear range of 0.01–300 ng mL^−1^, exhibiting a low detection limit of 6.3 pg mL^−1^. Increased multiplexing capability has been achieved through integration of aptamer‐magnetic particle capture matrix, CHA amplification, and a microfluidic chip electrophoresis for simultaneous detection of AFP and two other antigens, carcinoembryonic antigen (CEA) and carbohydrate antigen 125 (CA125), achieving low an LOD of 0.1, 0.2, and 0.15 pg mL^−1^, respectively.^[^ ^293^ ^]^ Toward point‐of‐care applications, a portable aptasensor system using a CRISPR‐powered personal glucose meter platform for quantitative detection of the AFP was demonstrated in spiked human serum samples with a detection sensitivity of 10 ng mL^−1^.^[^ ^295^ ^]^

Serum carcinoembryonic antigen (CEA) is elevated in various malignancies (e.g., colorectal cancer, medullary thyroid cancer, lung cancer, breast cancer, mucinous ovarian cancer, etc.), and CEA screening is part of various national and international surveillance guidelines.^[^ ^296^ ^]^ A large number of aptamer‐based sensors have been developed over the past decade, targeting sensitive and specific detection of CEA.^[^ ^297^, ^298^, ^299^, ^300^, ^301^, ^302^, ^303^, ^304^, ^305^, ^306^, ^307^ ^]^ For example, Shu et al. used two aptamers conjugated to magnetic beads and nickel hexacyanoferrate nanoparticles as a dual recognition system for chemiluminescent detection of CEA in spiked serum samples with an LOD of 0.092 pg mL^−1^.^[^ ^300^ ^]^ A lower LOD of 18.2 fg mL^−1^ was demonstrated using an electrochemical sensor in combination with HCR amplification and magnetic beads functionalized with a CEA‐specific aptamer.^[^ ^301^ ^]^ Label‐free microfluidic paper‐based electrochemical aptasensor was developed by Wang et al. using a nanocomposite‐modified electrode surface for parallel detection of CEA and neuron‐specific enolase (NSE) in clinical samples, with an LOD corresponding to 2 pg mL^−1^ for EA and 10 pg mL^−1^ for NSE.^[^ ^306^ ^]^ A high level of integration was achieved by Chakraborty et al. toward point‐of‐care device development using an electrochemical aptasensor for serum CEA detection, interfaced with a smartphone‐based handheld potentiostat.^[^ ^302^ ^]^ This sensor used a graphene/ZnO nanorod electrode and electrochemical impedance spectroscopy to achieve a detection limit of 1 fg mL^−1^.

#### 5.1.2 Detection of Circulating Tumor Cells

Circulating tumor cells (CTCs) are an important component of circulating targets, carrying substantial disease‐related molecular information and playing a key role in liquid biopsy.^[^ ^308^ ^]^ CTCs are associated with tumor progression,^[^ ^309^ ^]^ and an increasing number of clinical studies indicating that the enumeration of CTCs can serve as a predictor for early cancer diagnosis, therapeutic monitoring, prognosis, and recurrence^[^ ^310^ ^]^ have suggested the need to develop sensitive and specific CTC analytics. Combining the aptamer‐ligand pairs with amplification technologies has resulted in a number of CTC detection techniques.^[^ ^221^, ^308^ ^]^ For instance, a CHA amplification strategy for homogeneous visual and fluorescent detection of CTCs from clinical blood samples of patients with lung cancer was demonstrated using a double‐stranded DNA aptamer probe and quantum dots (QDs) as the nanoscale signal reporter.^[^ ^311^ ^]^ Under optimized conditions, the LOD of the fluorescence readout was found to be 3 cells/mL, with the assay allowing for the distinction of 100 cells/mL by simple visualization. Additionally, a nanopore‐sensing method with aptamer‐mediated amplification was tested to detect CTCs in clinical blood samples of breast cancer patients.^[^ ^312^ ^]^ However, the extraordinary rarity of CTCs in the bloodstream makes their detection a significant technological challenge. Often, a prerequisite to successful CTC detection is upfront isolation and purification. The combination of microfluidic technology with aptamer recognition for CTC trapping as well as controlled release is a promising avenue for efficient isolation of cancer cells from blood for downstream genomic or proteomic analysis.^[^ ^313^, ^314^, ^315^, ^316^, ^317^, ^318^ ^]^

A recent review by Sun et al. detailed progress in combining the power of microfluidic processing and aptamer recognition capacity for isolation, purification, as well as detection of CTCs.^[^ ^308^ ^]^ However, most of the reported detection technologies utilized model cells suspended in buffers^[^ ^321^, ^322^, ^323^ ^]^ with mixed cell populations^[^ ^324^, ^325^ ^]^ and require further development and validation with real samples for potential deployment and clinical translation. Toward CTC detection in complex fluids, Zhang et al. developed a novel biomimetic microfluidic system that integrates an aptamer specific to epithelial cell adhesion molecule (EpCAM) conjugated on functionalized iron‐oxide magnetic nanoclusters with a nickel‐patterned microfluidic device.^[^ ^326^ ^]^ Using a magnetically controlled microfluidic chip, the nanoclusters formed an in‐flow capture matrix for CTCs, allowing rapid processing of whole blood samples and downstream fluorescent detection of over 90% of the rare tumor cells (e.g., down to five cells in the 20 µL sample volume) within 20 min.

To improve aptamer stability and CTC capture efficiency, Peng et al. have proposed precise control over the orientation and valence of aptamers using programmable cubic DNA nanostructure aptamers immobilized at a microfluidic interface containing herringbone structures (Figure 9C,D).^[^ ^319^ ^]^ The proposed technology allowed successful isolation of CTCs from the peripheral blood of T‐cell leukemia patients and discrimination of T‐cell leukemia patients from healthy volunteers. Future integration of online detection could provide this system with the required capacity for potential translation to clinical applications. Toward this goal, Chen et al. proposed an automated microfluidic platform integrated with CTC‐specific aptamer‐bound field‐effect transistors (FET) sensor arrays and hydrodynamic cell trapping microstructures, enabling continuous CTC capture and simultaneous enumeration of up to 42 cells with a resolution of three cells.^[^ ^327^ ^]^ Before CTC detection, the whole blood sample was first processed on‐chip to lyse red blood cells, deplete leukocytes, and capture CTC on aptamer‐conjugated magnetic beads with a method developed by the same group.^[^ ^328^ ^]^ To increase the efficiency and accuracy of CTC capture, Zhao et al. developed a silicon nanowire interface grafted with multiple aptamers embedded within a microfluidic device comprising chaotic mixing structures.^[^ ^329^ ^]^ Following capture, the cells were fluorescently stained and imaged using a fluorescence microscope, demonstrating differential capture of CTCs for non‐small cell lung cancer patients, offering a potential solution for the selective enrichment of CTC subpopulations.

Using dual‐multivalent‐aptamer‐conjugated nanoprobes in concert with a microfluidic sorting and purification device, and downstream inductively coupled plasma mass spectrometry analysis, Zhang et al. demonstrated the manipulation and detection of a single CTC in whole blood within one hour.^[^ ^330^ ^]^ Recently, an on‐chip strategy for in situ CTC isolation and phenotyping combining size‐based microfluidic cell isolation from breast cancer patient blood with multiple spectrally orthogonal SERS aptamer nanovectors targeting distinct biomarkers on the cell surface was proposed to identify cancer subpopulations.^[^ ^331^ ^]^ Similarly, Gao et al. demonstrated a microfluidic chip with embedded lantern‐like bypass structures for capture and in situ heterogeneous phenotype analysis of single CTCs from whole blood samples of hepatocellular carcinoma patients using SERS‐aptamer nanotags for spectral recognition and fingerprinting.^[^ ^332^ ^]^ Multivalent SERS‐aptamer nanotags were optimized for stability and assay robustness, allowing multiplex detection of different cell‐surface biomarkers in situ from clinical specimens, suggesting strong potential toward clinical applications.

#### 5.1.3 Detection of Exosomes

Exosomes, extracellular vesicles released by cells, are found in the tumor microenvironment and are implicated in tumorigenesis by regulating angiogenesis, immunity, and metastasis. They serve as liquid biopsies and noninvasive biomarkers for cancer detection, diagnosis, and treatment.^[^ ^333^ ^]^ Several aptasensors for exosome detection have been developed in recent years. For instance, an electrochemical aptasensor for profiling HepG2 liver cancer exosomes was developed using a DNA nanotetrahedron (NTH) coupled with Au nanoparticles (NPs) and enzymatic signal amplification (Figure 9E).^[^ ^320^ ^]^ Additionally, a fluorescent aptasensor combining aptamers with aggregation‐induced emission (AIE) luminogens and graphene oxide as recognition elements was designed to detect exosomal tumor‐associated proteins in breast and prostate cancer patients.^[^ ^334^ ^]^ Toward a higher level of integration, a detachable microfluidic device with an electrochemical aptasensor was created for sequential analysis of cancerous exosomes from plasma samples of patients with breast cancer at different stages of the disease.^[^ ^335^ ^]^ These examples show that the application of aptamers for cancer exosome detection offers exciting opportunities to improve cancer detection, prognosis, and treatment monitoring, ultimately contributing to better patient outcomes and personalized medicine approaches.

Compared to circulating tumor cells and soluble tumor biomarkers, relatively fewer aptasensors have been developed for exosome detection. This disparity mainly arises from the intrinsic complexity of exosomes, which are nanosized vesicles (30–150 nm) with heterogeneous composition and surface marker expression depending on their cellular origin and isolation method. Such variability complicates both the identification of specific binding targets and the selection of high‐affinity aptamers through SELEX. Moreover, the limited availability of well‐validated exosome‐specific aptamers continues to constrain assay development and standardization. Recent reviews highlight that aptamer selection of extracellular vesicles is considerably more challenging than for purified proteins, and that the scarcity of suitable aptamers remains a major bottleneck for exosome biosensing.^[^ ^336^, ^337^, ^338^ ^]^

### 5.2 Cardiovascular Disease

**Figure 10:**
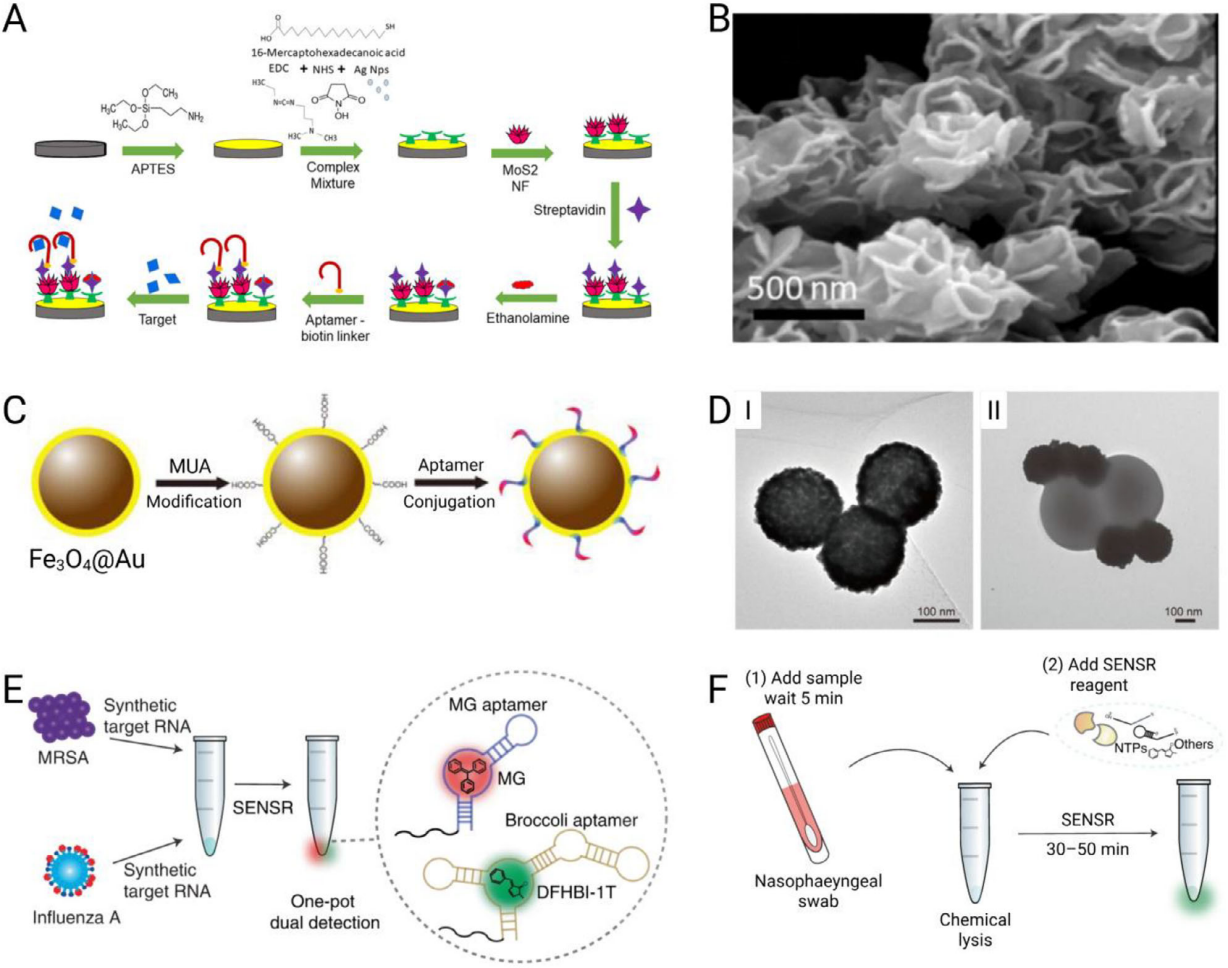
Aptamers applied to detecting A,B) cardiovascular disease, C,D) bacterial infections, and E,F) viral infections. A) Schematic illustration of the steps involved in surface modification of screen‐printed electrodes with aptamer‐tethered MoS_2_ nanoflower to recognize acute myocardial infarction biomarker. B) High magnification FESEM image of MoS_2_ nanoflower. Adapted with permission.^[^ ^341^ ^]^ Copyright 2020, John Wiley and Sons. C) Synthesis of aptamer‐modified Fe_3_O_4_@Au MNPs. D) Fe_3_O_4_@Au MNPs under (I) HRTEM and (II) TEM with *S. aureus* captured on the MNP surface. Adapted with permission.^[^ ^360^ ^]^ Copyright 2019, Elsevier. E) One‐pot dual detection of MRSA and influenza A. F) Schematics of SARS‐CoV‐2 detection from clinical samples by SENSR. Clinical samples treated by chemical lysis. Adapted with permission.^[^ ^366^ ^]^ Copyright 2020, Springer Nature. Abbreviations: APTES, (3‐Aminopropyl)triethoxysilane; DFHBI‐1T, (5Z)‐5‐[(3,5‐difluoro‐4‐hydroxyphenyl)methylene]‐3,5‐dihydro‐2‐methyl‐3‐(2,2,2‐trifluoroethyl)‐4H‐imidazol‐4‐one; MNP, Magnetic Nanoparticles; MoS_2_, molybdenum disulfide; MRSA, methicillin‐resistant *Staphylococcus aureus*; MUA, 4‐mercaptobenzoic acid (4‐MBA), ethanolamine,11‐mercaptoundecanoic acid; SENSR, Splint‐based One‐pot Isothermal RNA Detection.

The detection of cardiac biomarkers is used for diagnostics, prognostics, and cardiovascular risk assessment. For instance, cardiac troponin I (cTnI) is a recognized biomarker for the diagnosis of acute myocardial infarction (AMI) and related cardiovascular diseases.^[^ ^339^ ^]^ To date, various aptamers have demonstrated analytical potential for cTnl. Recently, an ultrasensitive electrochemical biosensor that combined a terminal deoxynucleotidyl transferase‐mediated signal amplification strategy with a cTnI aptamer has been demonstrated, achieving the detection level of cTnI at 40 pg mL^−1^ and good performance in different spiked biological samples (urine, saliva, serum).^[^ ^340^ ^]^ Vasudevan et al have deployed molybdenum disulfide (MoS_2_) nanosheets on the surface of the SPE electrode as a biointerface for cTnI aptamer immobilization. The electrochemical aptasensor exhibited high selectivity when human serum samples were used and exhibited an LOD of 1 fM (**Figure** **10**A,B).^[^ ^341^ ^]^

A ratiometric aptasensor for quantifying cTnI using SERS on a high‐density gold array functionalized with a highly specific cTnI aptamer was demonstrated with an LOD of 0.27 pg mL and performance in human serum comparable to ELISA.^[^ ^342^ ^]^ Chen et al. have developed an electrochemical aptasensor that leveraged aptamer target recognition, trans‐cleavage activity of CRISPR/Cas12a, as well as highly efficient separation capability of magnetic nanoparticles, to detect cTnI at an LOD of 10 pg mL^−1^ and high specificity in human serum.^[^ ^343^ ^]^ Expanded multiplexing capabilities using an impedimetric aptasensor were demonstrated using nanohybrids composed of nanodiamonds and hydrogen‐substituted graphdiyne functionalized with myoglobin and cTnI aptamers to achieve an LOD of 6.29 and 9.04 fg mL^−1^, respectively, with acceptable performance in human serum.^[^ ^344^ ^]^ Similarly, Grabowska et al. have reported multiplex detection of cardiac biomarkers in serum using aptamer‐based electrochemical sensors targeting brain natriuretic peptide and cardiac troponin I (cTnI).^[^ ^206^ ^]^

Higher level of multiplexing was demonstrated by Beduk et al. using a rapid immunodiagnostic sensor platform comprising nanostructured gold‐modified laser‐scribed graphene for simultaneous electrochemical detection of cTnl, cardiac troponin T (cTnT), and C‐reactive protein (CRP) from blood samples of 51 acute myocardial infarction patients with LODs of 2.58, 1.65, and 1.84 ng mL^−1^, respectively.^[^ ^345^ ^]^ These electrochemical aptasensors represent a step further toward multianalyte sensing of cardiac biomarkers. Toward the development of a point‐of‐care (POC) diagnostic platform, an amperometric aptasensor for the specific detection of cTnI was developed using disposable screen‐printed carbon electrodes coated with a carboxyethylsilanetriol‐modified graphene oxide derivative.^[^ ^346^ ^]^ The aptasensor was used to detect the cardiac biomarker in the 1.0–1.0 g mL^−1^ range, with a detection limit of 0.6 pg mL^−1^. Further advancement toward POC aptasensor deployment was demonstrated by Ma et al. by combining a mobile phone with a simple mobile software application and an electrochemical aptasensor comprising a screen‐printed carbon electrode for selective and sensitive cTnI detection in undiluted human serum samples with an LOD of 8.46 pg mL^−1^.^[^ ^347^ ^]^

### 5.3 Infectious Disease

Accessible diagnostic devices capable of detecting pathogenic microorganisms are crucial for preventing and treating infectious diseases. As witnessed during the COVID‐19 pandemic, there is a pressing demand for enhanced diagnostic tools, emphasizing attributes such as rapid detection, precision, cost‐effectiveness, and portability, thereby rendering detection strategies applicable even in resource‐limited environments. Over the past decade, nucleic acid aptasensors have shown potential to address this need, targeting a range of pathogens, including fungi, bacteria, viruses, and others.^[^ ^211^, ^348^, ^349^, ^350^, ^351^ ^]^

#### 5.3.1 Bacterial Infections

Aptamers have also been widely explored for the rapid and sensitive detection of bacterial pathogens, addressing the growing need for accessible and reliable diagnostic tools for bacterial infections.^[^ ^352^ ^]^ Diagnosing tuberculosis, an infectious bacterial disease that is most prevalent in third‐world countries, could especially benefit from the development of efficient, affordable, and accessible tests to allow prompt and effective management.^[^ ^353^ ^]^ Using an aptamer specific to the HspX antigen of *Mycobacterium tuberculosis* in concert with Aptamer Linked Immobilized Sorbent Assay (ALISA), Dhiman et al. have demonstrated successful detection of tuberculous meningitis in cerebrospinal fluid samples from patients.^[^ ^100^ ^]^ The same group successfully employed this aptamer for the detection of pleural tuberculosis with high sensitivity and selectivity, using ALISA as well as an electrochemical assay, providing translational potential to bridge the existing gap in TB diagnosis.^[^ ^354^, ^355^ ^]^

Another electrochemical aptasensor utilizing aptamer‐decorated gold nanoparticles anchored to carbon nanocomposite for signal amplification was demonstrated for rapid and sensitive determination of the *M. tuberculosis* MPT64 antigen in clinical serum samples.^[^ ^356^ ^]^ Using impedance spectroscopy and gold electrodes as substrates, Sypabekova et al. demonstrated successful detection of MPT64 antigen in serum samples with a limit of detection of 81 pMol for MPT64 and a reduction in the assay time from days/hours to 30 min, providing a promising prospect for TB diagnosis in resource‐limited settings.^[^ ^357^ ^]^ In addition, methodological evaluation and prospective clinical sample validation of aptasensors for other pathogenic bacteria, such as *Acinetobacter baumannii*, *E. coli*, *Salmonella enterica*, *Staphylococcus aureus* (Figure 10C,D), *Shigella sonnei*, *Clostridium difficile*, and others.^[^ ^358^, ^359^, ^360^, ^361^, ^362^, ^363^ ^]^

These advances demonstrate the versatility and diagnostic potential of aptamer‐based biosensors for detecting bacterial pathogens across a range of clinical and environmental contexts. Other studies demonstrating the integration of aptamers with nanomaterials,^[^ ^363^ ^]^ microfluidic systems,^[^ ^364^ ^]^ and electrochemical platforms^[^ ^365^ ^]^ have demonstrated the potential for the enhanced sensitivity, selectivity, and speed of bacterial detection required for moving aptasensor technologies closer to real‐world diagnostic implementation. Nonetheless, challenges remain in terms of clinical validation, large‐scale production, and performance consistency in complex biological samples. Continued interdisciplinary efforts combining aptamer engineering with molecular engineering, materials science, and translational research will be essential to realize the full potential of aptamer‐based bacterial diagnostics.

#### 5.3.2 Viral Infections

Biosensing strategies that employ aptamers for rapid and sensitive detection of viral infections have attracted a lot of attention in recent years^[^ ^367^, ^368^, ^369^, ^370^, ^371^, ^372^ ^]^, and their potential has been highlighted by a variety of strategies developed during the COVID‐19 pandemic. For example, a G‐quadruplex‐forming DNA aptamer against spike trimer antigen of SARS‐CoV‐2 was discovered, and its clinical utility was demonstrated using ALISA on 232 nasopharyngeal swab specimens for accurate SARS‐CoV‐2 diagnostics.^[^ ^373^ ^]^ A one‐step thermophoretic assay using polyethylene glycol and a fluorescently labeled aptamer specific to the spike protein of SARS‐CoV‐2 for direct quantitative detection of viral particles in spiked oropharyngeal swabs was demonstrated.^[^ ^374^ ^]^ The assay did not require pretreatment of viral particles and correctly identified SARS‐CoV‐2 positive oropharyngeal swab samples within 15 min, providing a promising avenue for fast and accurate screening of virus‐infected individuals.

Another assay enabling direct virus particle detection without the requirement for sample preparation was introduced by Bai et al., demonstrating an electrochemical impedance aptasensor for sensitive and selective detection of H1N1 influenza viral particles.^[^ ^375^ ^]^ An electrochemical impedance sensor consisting of a gold electrode functionalized with a dimeric DNA aptamer was utilized to detect wild‐type SARS‐COV‐2 virus and its alpha and delta variants with high sensitivity (one viral particle per µL) in saliva samples from patients in under 10 min without any further sample processing.^[^ ^376^ ^]^ A sample‐to‐answer sandwich‐type immunoassay using magnetism‐controlled microfluidic devices and aptamer‐conjugated magnetic particles was successfully employed for fluorescence‐based detection of Ebola virus with a detection limit of 4.2 ng mL^−1^.^[^ ^377^ ^]^ The whole detection process that consisted of sample mixing, separation, and signal acquisition was highly integrated, demonstrating potential for point‐of‐care applications. A portable two‐channel localized surface plasmon resonance detection system for sensitive and selective identification of SARS‐CoV‐2 spike protein S1 was recently demonstrated.^[^ ^378^ ^]^

Outside of COVID‐19, a noteworthy aptasensor for detecting Zika virus in human serum was developed ultizing an electrochemical approach. This biosensor is constructed using an aptamer nanoparticle heterolayer on an Au micro‐gap electrode, enabling highly sensitive detection of the virus (LOD of 38.14 pM).^[^ ^379^ ^]^ Toward multiplex viral detection, Saraf et al. developed a system by integrating a colorimetric aptasensor detection scheme using a sandwich‐type immunoassay utilizing gold and silver nanoparticles within a microfluidic device targeting Zika and chikungunya viral envelope proteins.^[^ ^380^ ^]^ A highly versatile one‐pot, ligation‐dependent isothermal reaction cascade producing an RNA aptamer that binds to a fluorogenic dye in the presence of a target RNA was demonstrated for a range of pathogens, including methicillin‐resistant *Staphylococcus aureus* (MRSA, *Vibrio vulnificus*, *Escherichia coli* O157:H7, Middle East respiratory syndrome‐related coronavirus (MERS‐CoV), and influenza A viruses, as well as SARS‐CoV‐2 (Figure 10E,F).^[^ ^366^ ^]^ Most recently, a new single‐step electrochemical detection method that uses single‐frequency impedance analysis for real‐time kinetic profiling of the interaction between a high‐affinity aptamer and an antigen on the viral surface was demonstrated in concert with machine learning for accurate diagnosis of SARS‐CoV‐2 and Influenza A viruses with a limit of detection less than 1100 copies/mL.^[^ ^381^ ^]^ By taking advantage of the high binding affinity of multimeric aptamers engineered to extract their target analytes from solution using three flexible arms and deliver them to electrode surfaces using a fourth non‐binding arm, the requirement for any washing or reagent addition steps was alleviated. Application of this technique to 37 human saliva samples for COVID‐19 diagnosis yielded both 100% sensitivity and 100% specificity.

The developments reviewed in this section demonstrate the potential of aptasensors as a powerful and versatile platform for medical diagnosis, with the potential to revolutionize healthcare by enabling rapid, sensitive, and specific detection of disease biomarkers in a cost‐effective and non‐invasive manner. Nevertheless, despite this progress, real‐world clinical applications of aptamer‐based biosensors are limited. For aptamer‐based assays to gain widespread acceptance within the industry, they must undergo thorough testing to validate their accuracy, reproducibility, and ease of use. One‐step assays and higher levels of integration and automation of aptasensor systems are key to alleviating the need for manual interventions for sample preparation, reagent transfer, and mixing. Continued research and innovation in aptamer technology and sensor development are likely to further enhance their utility in clinical practice.

#### 5.3.3 Parasitic and Fungal Diseases

Aptamer technology has begun to address important diagnostic gaps for parasitic and fungal pathogens, where antigenic variability, slow culture, and matrix interference often limit conventional assays. In malaria, multiple DNA aptamers have been developed against *Plasmodium* biomarkers, notably *Plasmodium falciparum* histidine‐rich protein II (PfHRP2) and parasite lactate dehydrogenase (PfLDH). A reusable, square‐wave voltammetry aptamer sensor targeting PfHRP2 quantified antigen in human serum, with an LOD of 3.73 nM, remained stable in serum buffers, demonstrating translational feasibility for point‐of‐care electrochemical readouts.^[^ ^382^ ^]^ Combining anti‐PfLDH aptamers in a DNA origami assembly preserved target‐specific binding with an LOD of 500 nM and illustrates how nanostructured scaffolds can be used in conjunction with aptamers to develop a serum‐stable diagnostic aptasensor, a strategy relevant to low‐parasitemia screening.^[^ ^383^ ^]^ Broader overviews emphasize that aptamers to *Plasmodium* antigens are increasingly integrated with multiplexable sensor platforms and paper‐based microfluidics to reduce cost and cold‐chain dependence for malaria diagnostics.^[^ ^384^ ^]^

For fungal pathogens, aptamers offer specific recognition without antibodies and can be formatted for tissue histology or rapid sensing. In candidiasis, the RNA aptamer Ca‐apt‐1 specifically recognized *Candida albicans* in paraffin‐embedded infected rat tongue sections with staining performance comparable to, or exceeding, a polyclonal antibody, supporting use as an immunohistochemical probe for clinical specimens.^[^ ^385^ ^]^ Beyond *Candida*, aptamer efforts span other fungi: DNA aptamers against the Asp f 1 allergen of *Aspergillus fumigatus* (a major invasive mold) demonstrated high‐affinity, selective binding, and recent reviews highlight β‐D‐glucan (BDG)‐binding aptamers as pan‐fungal surrogates for invasive disease detection and monitoring.^[^ ^386^, ^387^ ^]^

Aptamers have also been explored for neglected parasitic infections. In Chagas disease, Trypomastigote excreted‐secreted antigen (TESA) secreted by the *Trypanosoma cruzi* trypomastigote form were detected in plasma using aptamer probes in a non‐PCR, non‐serological format, suggesting utility for therapy monitoring and early diagnosis.^[^ ^388^ ^]^ For leishmaniasis, DNA aptamers recognizing *Leishmania infantum* histone H2A showed specific binding in ELONA and blot assays, laying the groundwork for sensor integration and species‐level discrimination.^[^ ^389^ ^]^ Collectively, these studies indicate that aptamers can (I) directly target pathogen proteins or cells, (II) report pan‐fungal markers such as BDG, and (III) interface with electrochemical or visual readouts to achieve rapid, sensitive, and potentially low‐cost diagnostics suited to decentralized testing. Continued priorities include rigorous clinical validation in endemic settings, standardization of sample preparation, and manufacturable device formats to translate promising prototypes into routine parasitic and fungal diagnostics.^[^ ^390^, ^391^ ^]^ A curated selection of aptasensors for medical diagnostic applications is shown in **Table** **4**.

**Table 4:** Selected representative aptasensor diagnostic platforms for various medical diagnostic applications.

	Disease	Biomarker Target	Detection Strategy	Limit of Detection	Ref.
Cancer	Prostate cancer	PSA	Colorimetric aptasensor using cationic polymer and poly‐adenine aptamer on gold nanoparticles	20 pg mL^−1^	[247]
	Prostate cancer	PSA	Impedimetric dual‐recognition aptasensor (nanostructured gold electrodes)	0.26–62.5 ng mL^−1^	[250]
	Breast cancer	HER2	Electrochemical impedance aptasensor on gold screen‐printed electrodes	170 pgmL^−1^	[260]
	Multiple cancers	MUC1	Dual‐mode SERS‐colorimetric aptasensor with magnetic separation	0.1 U mL^−1^	[266]
	Cervical Cancer	OPN & VEGF	Paper‐based multiplex colorimetric/SERS lateral flow aptasensor	OPN: 10 pg mL^−1^ VEGF: 0.8 pg mL^−1^	[280]
	Liver Cancer	AFP, CEA, & CA125	Microfluidic chip with CHA amplification	AFP: 0.1 pg mL^−1^ CEA: 0.2 pg mL^−1^ CA125: 0.15 pg mL^−1^	[293]
	Liver cancer	AFP	Enzyme‐free fluorescence aptasensor with CHA amplification	0.033 ng mL^−1^	[285]
	Various Cancers	CEA & NSE	Paper‐based electrochemical aptasensor	CEA: 2 pgmL^−1^ NSE: 10 pg mL^−1^	[306]
	Lung Cancer	CTC	CHA fluorescence aptasensor with QDs	3 cells/mL	[311]
	Breast/Prostate Cancer	Exosomes	Fluorescent aptasensor with AIE luminogens and graphene oxide	Not specified	[334]
Cardiovascular Diseases	Cardiovascular diseases including AMI	cTnI	MoS_2_ nanosheet electrochemical aptasensor	1 fM	[341]
	Cardiovascular diseases including AMI	Myo & cTnI	Impedimetric nanohybrid aptasensor	Myo: 6.29 fg mL^−1^ cTnI: 9.04 fg mL^−1^	[344]
	Cardiovascular diseases including AMI	cTnI, cTnT, CRP	Electrochemical aptasensor (laser‐scribed graphene)	cTnI: 2.58 ngmL^−1^ cTnT: 1.65 ng mL^−1^ CRP: 1.84 ng mL^−1^	[345]
Infectious Diseases	Tuberculosis	HspX antigen	ALISA and electrochemical aptasensor	Not specified	[100, 354, 355]
	Tuberculosis	MPT64 antigen	Electrochemical impedance aptasensor	81 pM	[357]
	Influenza A (H1N1)	Viral particle	Electrochemical impedance aptasensor	Not specified	[375]
	Influenza A & SARS‐CoV‐2	Viral antigens	Electrochemical impedance aptasensor + machine learning	< 1100 copies/mL	[381]
	SARS‐CoV‐2	Viral particle	Electrochemical impedance aptasensor (dimeric DNA aptamer)	1 viral particle/µL	[376]
	Ebola Virus	Viral particle	Magnetic microfluidic fluorescence aptasensor	4.2 ng mL^−1^	[377]
	Zika Virus	Viral particle	Electrochemical aptasensor (nanoparticle heterolayer on Au micro‐gap)	38.14 pM	[379]
	Malaria	PfHRP2	Square‐wave voltammetry electrochemical aptasensor	3.73 nM	[382]
	Malaria	PfLDH	DNA origami‐assembled aptasensor	500 nM	[383]
	Candidiasis	*Candida albicans*	RNA aptamer (Ca‐apt‐1) for immunohistochemistry	Not applicable	[385]
	*Aspergillus fumigatus*	Asp f 1 allergen	DNA aptamer affinity assay	Not specified	[386]
	Chagas Disease	TESA	Aptamer‐based non‐PCR, non‐serological detection	Not specified	[388]
	Leishmaniasis	Histone H2A (*L. infantum*)	ELONA and blot aptamer assay	Not specified	[389]

Abbreviations: AFP, alpha‐fetoprotein; AIE, aggregation‐induced emission; ALISA, aptamer‐linked immobilized sorbent assay; AMI, acute myocardial infarction; CA125, carbohydrate antigen 125; CEA, carcinoembryonic antigen; CHA, catalytic hairpin assembly; CRP, C‐reactive protein; CTC, circulating tumor cell; cTnI, cardiac troponin I; cTnT, cardiac troponin T; ELONA, enzyme‐linked oligonucleotide assay; HER2, human epidermal growth factor receptor 2; HspX, heat shock protein X; MoS_2_, molybdenum disulfide; MUC1, Mucin‐1; Myo: myoglobin; NSE, neuron‐specific enolase; OPN, osteopontin; PfHRP2, *Plasmodium falciparum* histidine‐rich protein II; PfLDH, *Plasmodium falciparum* lactate dehydrogenase; PSA, prostate‐specific antigen; QD, quantum dot; SARS‐CoV‐2, severe acute respiratory syndrome coronavirus 2; SERS, surface‐enhanced Raman spectroscopy; TdT, terminal deoxynucleotidyl transferase; TESA, trypomastigote excreted‐secreted antigen; VEGF, vascular endothelial growth factor.

## 6 Non‐Medical Sensing Applications

The global demand for reliable food safety methods is surging, propelled by the increasing public awareness of food quality. In this context, food safety and authenticity are thus of utmost importance. Food safety detection requires accurate identification of organic and nonorganic sources, species type, undisclosed components, and geographical indications. Conventional PCR and ELISA, while precise, often require extensive sample preparation and skilled operators, making the authentication process challenging and time‐consuming.^[^ ^392^ ^]^

In the field of environmental monitoring, the detection of heavy metals (HMs) and rare earth elements (REEs) is a pressing issue. While various methods like (bio)sorbents and extraction techniques have been developed, they come with significant drawbacks.^[^ ^393^, ^394^, ^395^, ^396^ ^]^ These methods often require on‐site and routine monitoring, involve complex sample pretreatment, and use costly equipment. Moreover, the use of adsorbents/solvents may introduce additional environmental pollutants, making the need for biosensors even more evident.^[^ ^393^, ^394^, ^395^, ^396^ ^]^

On the other hand, the rapid and precise identification of drug misuse holds critical importance in forensic science and criminology. Presumptive testing of drugs in confiscated samples plays a key role in the application of laws against the possession, production, and distribution of illegal substances. Additionally, the early detection of drugs in biological samples is essential due to the fast pharmacokinetics and pharmacodynamics (PK/PD) associated with these substances.^[^ ^397^ ^]^ Nonetheless, many of the methods currently employed by law enforcement agencies have notable limitations. Gas chromatography paired with mass spectrometry (GC‐MS) and liquid chromatography coupled with mass spectrometry (LC‐MS) are regarded as the ′gold standard' in forensic drug analysis due to their exceptional sensitivity, selectivity, and reliability.^[^ ^398^, ^399^, ^400^ ^]^ However, these methods come with prohibitive costs and may not be suitable for high throughput because of their lengthy processing times.^[^ ^401^, ^402^ ^]^ Chemical spot tests, while quick, economical, and easy to implement, lack specificity.^[^ ^403^, ^404^ ^]^ Handheld Raman spectrometers are effective for analyzing uncontaminated drug samples, but their performance diminishes with mixed street opioids, often yielding ambiguous results.^[^ ^405^, ^406^ ^]^ Similarly, lateral‐flow immunoassays are proficient at identifying conventional opioids such as morphine and codeine but fail to provide the necessary specificity and sensitivity for detecting newer fentanyl derivatives.^[^ ^407^ ^]^

Overall, there is a strong interplay between the need for precise, reliable detection methods and the inherent challenges of adapting these technologies to diverse applications. Within this framework, aptamers emerge as one of the most versatile biosensing probes. Notable for their ease of synthesis and chemical simplicity compared to antibodies, aptamers offer superior stability and adaptability. These attributes make them highly effective for identifying environmental pollutants and enhancing environmental monitoring efforts.^[^ ^204^, ^408^ ^]^ Moreover, ongoing advancements in engineering of aptamer selection techniques, such as SELEX, have expanded the library of available aptamers for sensor development.^[^ ^409^, ^410^ ^]^

Aptasensors offer a remarkable solution for on‐site, real‐time detection, providing unmatched sensitivity and specificity. These sensors utilize the unique binding properties of aptamers to create detection platforms that can identify even trace amounts of contaminants, significantly improving monitoring efforts and preventive measures.^[^ ^204^, ^205^, ^408^ ^]^ The integration of aptamers into biosensing platforms has led to the development of various detection modalities, including colorimetric,^[^ ^279^, ^411^, ^412^ ^]^ fluorescent,^[^ ^413^ ^]^ electrochemical,^[^ ^414^ ^]^ and surface plasmon resonance (SPR) sensors.^[^ ^415^ ^]^ Each of these methods leverages the distinctive properties of aptamers to enable the direct or indirect detection of target molecules, facilitating applications in environmental surveillance, food quality control, and forensic analysis, and a curated selection of some of these non‐medical sensing applications are shown in **Table** **5**.^[^ ^205^ ^]^ Similar to the previous section, in this section, we highlight and discuss the most significant examples that have shaped progress in the area of non‐medical sensing.

**Table 5:** Summary of aptamer‐based detection approaches and applications in non‐medical sensing at the cell, molecular, and atomic/ionic scale.

	Target	Detection Strategy	Application	Limit of Detection	Ref.
Cell sensing	*Salmonella*	Colorimetric/fluorescent dual‐mode detection using DNA‐nanotriangle multivalent aptamer	Food safety	Colorimetric detection: 316 CFU/mL Fluorescent detection: 60 CFU/mL	[443]
	*S. aureus, E. coli*, *P. aeruginosa*	Multiplex aptamer‐based fluorescence assay	Food safety	*S. aureus*: 8 CFU/mL *E. coli*: 6 CFU/mL *P. aeruginosa*: 5 CFU/mL	[444]
	*Salmonella*	"Signal‐on" electrochemical DNA biosensor	Environmental monitoring and food safety	8 CFU/mL	[445]
	*S. aureus*	FRET‐based aptasensor with AuNPs and UCNPs	Food safety	10.7 CFU/mL	[446]
	*E. coli* O157:H7	Surface‐enhanced Raman spectroscopy	Food safety	Pure culture: ≈10 CFU/mL Ground beef: ≈100 CFU/mL	[358]
	*Salmonella typhimurium*	AuNPs colorimetric sensor based on dual aptamers	Food safety: point‐of‐care testing (POCT) in milk samples	Pure culture: 33 CFU/mL Spiked milk: 95 CFU/mL	[447]
	*Legionella pneumophila*	Cell‐SELEX technique using aptamers	Environmental monitoring: detection of *Legionella* in water systems	Not specified	[448]
	*L. pneumophila*	SPRi‐based titration assay using aptamers	Environmental monitoring: detection of *Legionella* in water systems	104.4 CFU/mL	[449]
Molecular (including biological macromolecules) sensing	Adenosine	Colorimetric sensing using aptamer‐functionalized AuNPs	Detection of adenosine for forensic and clinical toxicology	1.5 × 10^4^ ng mL^−1^	[450]
	Cocaine	Colorimetric sensing using aptamer‐functionalized AuNPs	Detection of cocaine for forensic and clinical toxicology	9.1 × 10^4^ ng mL^−1^	[450]
	Fentanyl and its analogues	Colorimetric, fluorescent, and electrochemical sensors	Forensic analysis, medical diagnostics, and public safety for rapid, accurate identification and quantification of fentanyl and its analogues in seized substances and potentially in biological samples.	E‐AB sensor: 5.05 ng mL^−1^ (specific LODs for other sensors not explicitly mentioned in the summary)	[451]
	Caffeine and its demethylated analogues (theophylline, theobromine, and paraxanthine)	Structure‐switching fluorescent aptasensor	Detection of caffeine and its analogues in beverages and potentially in biological samples	Caffeine in buffer: 233 ng mL^−1^	[421]
	Ochratoxin A (OTA)	ECL aptasensor based on CdSe/CdS QDs	Food safety: Lily and rhubarb samples	0.89 ng mL^−1^	[452]
	Ochratoxin A (OTA)	Bipolar electrode‐ECL biosensor	Food safety: detection of OTA in grains (rice, wheat, corn, sorghum, barley, buckwheat)	0.003 ng mL^−1^	[453]
	Aflatoxin B1 (AFB1)	ECL aptasensor based on gold nanorods/graphene QDs‐modified poly (indole‐6‐carboxylic acid)/flower‐Au nanocomposite	Food safety: detection of AFB1 in peanuts, maize, and wheat	0.00375 ng mL^−1^	[454]
	Aflatoxin M1 (AFM1)	Ru@COF‐LZU1 micro‐reactor‐based biosensor with confinement‐enhanced ECL	Food safety: detection of AFM1 in defatted milk samples	9 × 10^−6^ ng mL^−1^	[455]
	Microcystin‐LR (MC‐LR)	B N co‐doped graphene synergistically catalyzed ZnO QDs with amplified cathodic ECL	Fabricating a microcystin‐LR aptasensor for ecological environment safety and human health	3 × 10^−5^ ng mL^−1^	[456]
	Zearalenone (ZEN)	NGQDs‐NH_2_‐Ru@SiO_2_ luminophore‐based self‐enhanced ECL aptasensor	Monitoring ZEN in corn flour for food safety	1 × 10^−6^ ng mL^−1^	[457]
	Lincomycin	ECL aptasensor using SnO_2_/chitosan/g‐C_3_N_4_ nanocomposite	Detection of lincomycin in pharmaceutical and food samples	0.028 ng mL^−1^	[458]
	Tetracycline	PEC aptasensor based on Au/BiOI composites	Detection of tetracycline residues in wastewater for environmental monitoring and protection	2 × 10^−4^ ng mL^−1^	[459]
	Lincomycin (Lin)	ECL aptasensor based on Ag_3_PO_4_‐Ti_3_C_2_ nanohybrids	Detection of Lin in milk and water samples, showcasing the sensor's potential for real‐world applications in food safety and environmental monitoring	25.1 ng mL^−1^	[460]
	Chlorpyrifos	ECL aptasensor based on resonance energy transfer between MoS_2_/CdS nanospheres and Ag/CQDs	Detection of chlorpyrifos in fruit and vegetable samples, offering a new approach for agricultural and environmental sample analysis	1.23 × 10^−7^ ng mL^−1^	[461]
	Malathion	ECL aptasensor based on CdTe QDs@NH_2_‐MIL‐88(Fe) for signal amplification	Detection of Malathion in water samples, contributing to the monitoring of organophosphorus pesticide contamination	3 × 10^−7^ ng mL^−1^	[462]
	Malathion	Supersensitive ECL aptasensor utilizing ATO/TiO_2_ and AgNPs	Rapid detection of Malathion residues in environmental samples and food control, particularly in vegetables	2.5 × 10^−5^ ng mL^−1^	[463]
	Anatoxin‐a (ATX‐a)	DNAzyme‐based dual‐stimuli responsive ECL resonance energy transfer platform utilizing ATO/TiO_2_ and AgNPs	Environmental monitoring and water safety: ultrasensitive detection of ATX‐a in real water samples	0.34 ng mL^−1^	[464]
	Bisphenol A (BPA)	Aptamer‐based fluorescent method using nonconjugated AuNPs and CdTe QDs	Detection of BPA in environmental samples, particularly in drinking water	1.86 ng mL^−1^	[465]
	Murine Norovirus (MNV)	NanoZyme aptasensor	Viral detection in food and environmental samples	200 viruses/mL	[466]
Atomic/Ionic sensing	Mercury (Hg^2+^) and Lead (Pb^2+^) ions	Electrochemical aptasensor using M‐shaped functional DNA complexes	Food safety and environmental monitoring	Pb^2+^: 2.0 ng mL^−1^ Hg^2+^: 0.5 ng mL^−1^	[467]
	Lead ions (Pb^2+^)	Paper‐based fluorescent aptasensor	Food safety and environmental monitoring	1.3 ng mL^−1^	[468]
	Lead ions (Pb^2+^)	GQDs as the fluorophore and graphene oxide (GO) as the quenching platform	Environmental monitoring and safety assessments	0.1 ng mL^−1^	[469]
	Arsenite ions (As^3+^)	Graphene field‐effect transistors	Environmental monitoring (detecting As^3+^ in water)	0.02 ng mL^−1^	[470]
	Arsenite ions (As^3+^)	Aptamer‐AuNPs‐based colorimetric assay with a smartphone‐coupled optical unit	Environmental monitoring (detecting As^3+^ in contaminated soil)	Aqueous samples: 14.44 ng mL^−1^ Feld soil samples: 1.97 ng mL^−1^	[471]
	Cadmium ions (Cd^2+^)	Aptamer functionalized AuNPs based on a smartphone‐based colorimetric system	Environmental monitoring: assessing the contamination of water	1.12 ng mL^−1^	[472]
	Chromium (Cr^3+)^ and Chromium (Cr⁶⁺) ions	DNAzyme fluorescence signaling upon Cr^3+^ detection	Environmental monitoring of water for Cr^3+^ and Cr^6+^	Cr^3+^: 3.64 ng mL^−1^ Cr^6+^: 7.28 ng mL^−1^	[473]
	Cesium ions (Cs^+^)	Iridium(III) complex that enhances luminescence in the presence of G‐quadruplex DNA structures induced by Cs^+^ ions.	Environmental monitoring and safety: detecting Cs^+^ in areas affected by nuclear accidents	13 ng mL^−1^	[474]
	Mercury (Hg^2+^) and Lead (Pb^2+^) ions	Electrochemical sensing using DNA aptamers labeled with ferrocene (or methylene blue) and thiol groups at their 5′ and 3′ termini, leveraging target‐induced aptamer conformation change to modulate electron transfer and signal output	environmental monitoring, specifically for the rapid and sensitive detection of toxic heavy metals in water	Hg^2+^: 0.1 ng mL^−1^ Pb^2+^: 0.1 ng mL^−1^	[475]
	Nickel ions (Ni^2+^)	Small DNA aptamers that bind specifically and tightly to Ni^2+^, differentiating from similar ions like Co^2+^. The binding is confirmed through isothermal titration calorimetry	Environmental monitoring, specifically for the adsorption and detection of Ni^2+^ in wastewater	1409 ± 264 ng mL^−1^	[476]
	Copper ions (Cu^2+^)	Specific aptamers identified through SELEX for selective binding to copper ions	Environmental remediation and resource recovery (recovery of copper from aqueous solutions, including acidic mine drainage)	0.00116 ng mL^−1^	[477]

This table provides a comprehensive overview of various aptamer‐based detection methods for a range of targets, including bacteria, toxins, antibiotics, pesticides, and heavy metals. Each entry details the target name (Specifies the biological or chemical entity being detected), detection approach (Describes the method or technology used for detection), specific applications (Indicates the practical application or field of use, such as food safety, environmental monitoring, forensic analysis, and medical diagnostics), and the limit of detection (LOD; The minimum concentration of the target that can be reliably detected), highlighting the versatility and sensitivity of aptamers in different contexts. LOD values have been standardized, where possible, for comparison.

Abbreviations: AuNPs, gold nanoparticles; ECL, electrochemiluminescence; FRET, fluorescence resonance energy transfer; GQDs, graphene quantum dots; PEC, photoelectrochemical; QDs, quantum dots; Ru@COF‐LZU1, refers to the integration of ruthenium(II) tris(2,2'‐bipyridyl) (Ru(bpy)_3_^2+^) with the covalent organic framework LZU1; SELEX, systematic evolution of ligands by exponential enrichment; SPRi, surface plasmon resonance imaging; UCNPs, upconverting nanoparticles.

### 6.1 Food Safety Monitoring

The application of aptamer‐based biosensors in food safety monitoring is still in its early stages, but several promising developments have been reported. For instance, aptamers have been developed to detect melamine, a harmful adulterant found in milk products. These aptamer‐based assays for melamine detection have shown exceptional sensitivity and specificity, presenting a rapid and reliable alternative to traditional methods. Typically, these assays involve the use of gold nanoparticles or similar materials that generate a visible color change in the presence of the target molecule, enabling straightforward and quick detection.

**Figure 11:**
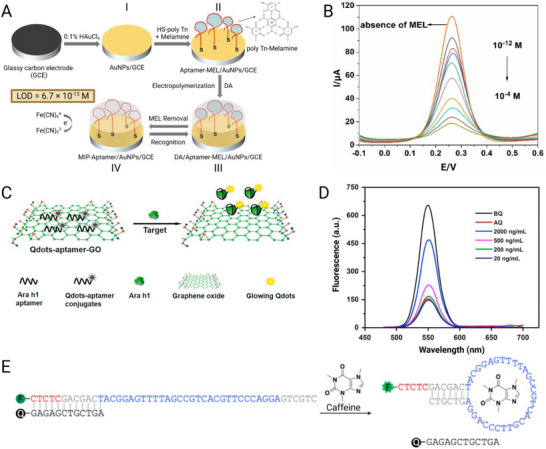
A) Fabrication of MIP‐Aptamer/AuNPs/GCE for melamine detection: I) AuNPs electrodeposited on a glassy carbon electrode (GCE) for a high surface area, II) Aptamer‐MEL complex immobilized on AuNPs/GCE via Au‐S bonding, III) Electropolymerization of dopamine (DA) forming molecularly imprinted surfaces, IV) Removal of melamine templates to create recognition cavities. B) Differential pulse voltammetry (DPV) profiles of MIP‐Aptamer/AuNPs/GCE incubated with melamine concentrations (10^−12^ M to 10^−4^ M), showing reduced current with increased melamine. Adapted with permission.^[^ ^416^ ^]^ Copyright 2021, Elsevier. C) Schematic of the Qdots‐aptamer‐GO quenching system used for peanut allergen (Ara h 1) detection. Fluorescence quenching by GO is reversed upon target binding, enabling allergen identification. D) Fluorescence emission spectra of a quantum‐dot‐aptamer‐graphene oxide (Qdots‐aptamer‐GO) sensing system used for detecting the peanut allergen Ara h 1 at various concentrations. As Ara h 1 concentration increases, the fluorescence intensity in the recovery spectra rises proportionally. Adapted with permission.^[^ ^419^ ^]^ Copyright 2016, Elsevier. E) Structure‐switching fluorescent aptamer biosensor for caffeine detection. The binding of caffeine to the Caff203 aptamer causes the release of a quencher strand, resulting in fluorescence enhancement. Adapted with permission.^[^ ^421^ ^]^ Copyright 2022, American Chemical Society. Abbreviations: AQ, after quenching (the fluorescence decrease after interaction with graphene oxide); BQ, before quenching (the initial fluorescence intensity of Qdots‐aptamer probes); MEL, melamine.

An example of using aptamers for detecting melamine in food products is the development of a highly sensitive molecularly imprinted electrochemical aptasensor (MIEAS).^[^ ^416^ ^]^ This advanced sensor combines the unique selectivity of molecularly imprinted polymers (MIPs) with the high affinity of aptamers to achieve specific and sensitive detection of melamine (**Figure** **11**A). This aptasensor employs gold nanoparticles (AuNPs) synthesized via sodium citrate reduction. The MIPs, formed by electropolymerization of dopamine with polythymine aptamers as functional monomers and melamine as the template, provide high specificity. The sensor demonstrated a linear detection range from 10^−12^ M to 10^−4^ M and a detection limit of 6.7 × 10^−13^ M, as shown in Figure 11B.

In another recent work by Yujing Ma et al., a DNA walker‐mediated SERS sensing technique was developed.^[^ ^417^ ^]^ This method employs melamine‐specific aptamers as identification probes integrated with gold‐coated magnetic nanoparticles (AuNPs@MNPs) and small gold nanoparticles (AuNPs@MBA) to amplify the Raman signal. The biosensor can quantitatively detect melamine within a concentration range of 0.001–500 mgkg^−1^, achieving a detection limit of 0.001 mgkg^−1^ and an enhancement factor of 2.3 × 10^7^. This technique is both sensitive and rapid, making it suitable for on‐site detection of melamine in milk and dairy products.

In addition to melamine, aptamers have been used to detect pathogenic bacteria in food samples, providing results in a fraction of the time required by traditional microbiological methods, making them valuable tools for ensuring food safety in real‐time. Many reviews and research articles have been published on the use of aptasensors for bacteria sensing,^[^ ^418^ ^]^ and some recent examples are cited in Table 5.

Aptamer‐based sensors have also been shown to be able to detect food allergens with high sensitivity, enabling the identification of trace amounts that might be missed by conventional methods.^[^ ^419^, ^420^ ^]^ For instance, Weng et al. developed a microfluidic biosensor using graphene oxide and aptamer‐functionalized quantum dots for peanut allergen detection (Figure 11C).^[^ ^419^ ^]^ Figure 11D demonstrates the biosensor's high specificity and sensitivity in detecting Ara h 1, the major peanut allergen, by exploiting fluorescence quenching and recovery mechanisms. The system achieved a detection limit of 56 ng mL^−1^, as shown in their standard calibration curve. The study highlights the effectiveness of graphene oxide as a fluorescence quencher and its potential for enhancing the performance of aptamer‐based biosensors.

To further illustrate the adaptability of aptamer‐based biosensors, Huang and Liu demonstrated the versatility of aptamers by isolating four DNA aptamers with high specificity for caffeine and its analogues (Figure 11E).^[^ ^421^ ^]^ Their study highlighted the aptamers' ability to distinguish caffeine from closely related molecules like theophylline, theobromine, and paraxanthine. They further developed a structure‐switching fluorescent sensor with a detection limit of 1.2 µM caffeine, successfully validating its performance in real samples such as beverages and human serum.

Despite their potential, aptamer‐based biosensors face several challenges that must be addressed to fully realize their capabilities in food safety. One of the main challenges is the identification and validation of reliable biomarker molecules that can serve as targets for aptamer binding. This process requires extensive research to identify markers that are specific to certain contaminants or adulterants and that remain stable under various conditions.

Another challenge is the development of robust and user‐friendly sensor platforms that can be easily used in real‐world settings, such as food processing facilities or at points of sale. While laboratory‐based studies have demonstrated the effectiveness of aptamer‐based sensors, translating these findings into practical applications requires the development of portable, cost‐effective, and easy‐to‐use devices.

Future research should focus on expanding the range of targets that can be detected by aptamer‐based sensors, improving the sensitivity and specificity of these devices, and integrating them with advanced technologies such as machine learning and data analytics. Developing databases to store biochemical profiles of food and applying machine learning algorithms can accelerate the identification of reliable biomarkers for food safety, enhancing the efficiency and accuracy of the detection process.^[^ ^422^ ^]^

### 6.2 Environmental Monitoring

The contamination of water sources and the shortage of freshwater resources are pressing international concerns, posing severe medical, economic, and environmental challenges. Annually, waterborne diseases claim over two million lives, a direct consequence of contamination by pathogenic bacteria, highlighting a critical interconnection between water and soil pollution.^[^ ^204^ ^]^ This complex relationship significantly impacts human health, the environment, and industries reliant on agriculture and aquaculture, necessitating the development of precise detection methods for contaminants, including bacteria, toxins, and heavy metals. Simultaneously, heavy metal pollution exacerbates the global freshwater crisis, underscoring the urgent need for innovative, green, and cost‐effective solutions for detecting and eliminating heavy metal ions. Emerging strategies, including the use of nanomaterials, polymers, porous materials, biomaterials, and notably aptamers, offer promising avenues for real‐time detection and efficient removal of heavy metals, thanks to their high selectivity, low detection limits, and resistance to interference.^[^ ^203^, ^423^, ^424^ ^]^ In this subsection, we will explore the latest advancements in environmental monitoring, with a specific focus on bacteria sensing and the detection and remediation of heavy metals in water, demonstrating the pivotal role of cutting‐edge aptamer biosensing in addressing these environmental challenges.^[^ ^425^ ^]^

**Figure 12:**
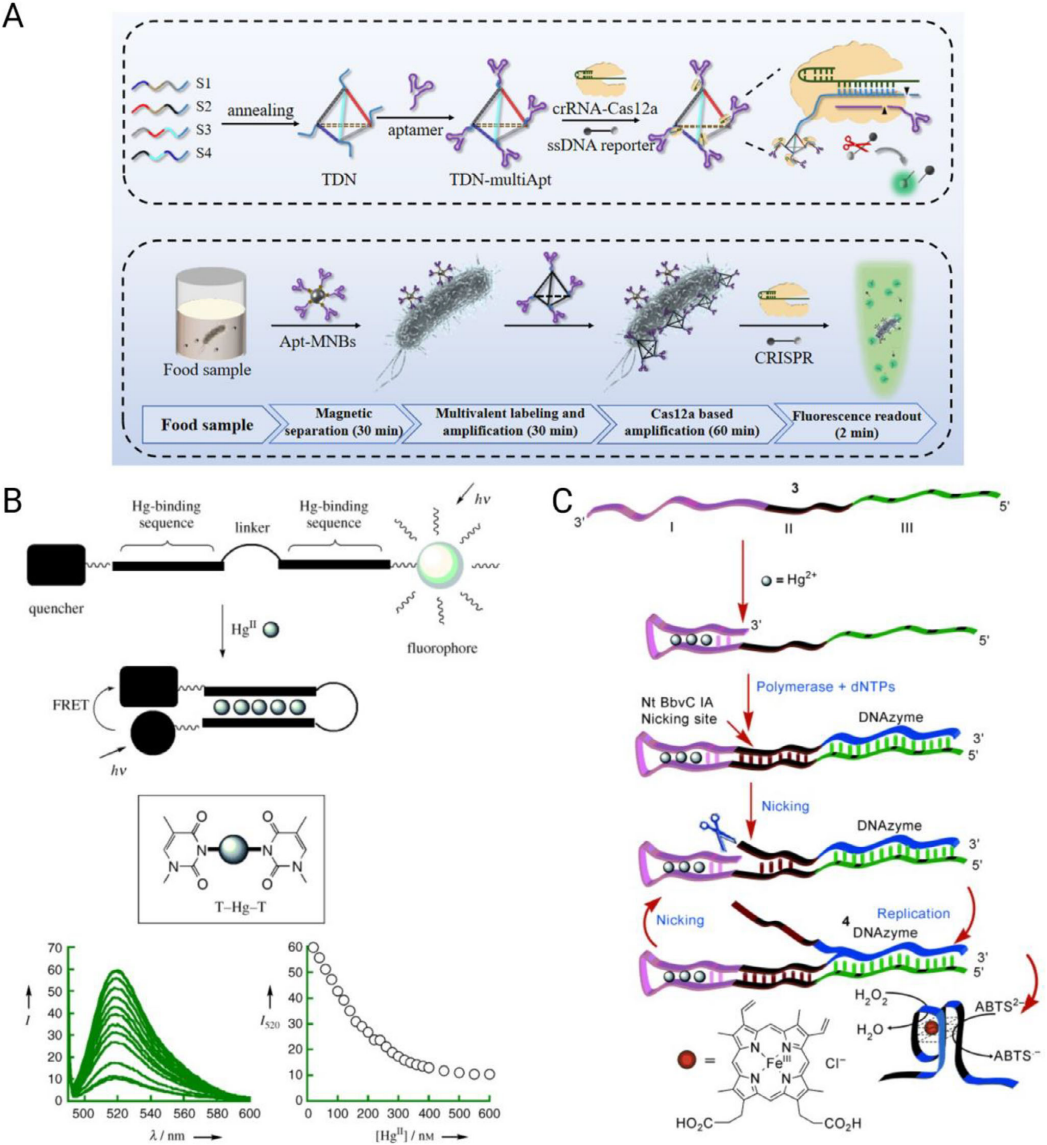
A) Schematic representation of TDN‐multiApt assembly and its application in an amplification‐free CRISPR/Cas12a assay for sensitive detection of *Salmonella*. The dual‐functional tetrahedral DNA nanostructure enhances aptamer avidity and signal amplification through multivalent interactions. Adapted with permission.^[^ ^426^ ^]^ Copyright 2023, American Chemical Society. B) Schematic illustration of a DNA sensor where Hg^2+^‐mediated T‐Hg‐T pairing induces a hairpin structure in D‐ODNF, resulting in fluorescence quenching. Fluorescence intensity decreases with increasing Hg^2+^ concentrations (0–600 nM). Adapted with permission.^[^ ^429^ ^]^ Copyright 2004, John Wiley and Sons. C) Design and operation of a DNA‐based analytical machine for Hg^2+^ detection. The machine employs a track with multiple DNAzyme synthesis cycles triggered by Hg(II), facilitating sensitive colorimetric analysis. Adapted with permission.^[^ ^430^ ^]^ Copyright 2008, John Wiley and Sons.

A notable example of innovation in this field is the work by Qiao et al. (**Figure** **12**A), who developed a CRISPR/Cas12a assay combined with a tetrahedron multivalent aptamer (TDN‐multiApt) for detecting *Salmonella*.^[^ ^426^ ^]^ This system enhances aptamer avidity and includes CRISPR/Cas12a targeting fragments for signal amplification. In this mechanism, the CRISPR/Cas12a complex, guided by a CRISPR RNA (crRNA), recognizes specific DNA sequences on the tetrahedral scaffold, activating Cas12a's collateral cleavage activity toward nearby single‐stranded DNA reporters. This results in a strong fluorescent signal without requiring nucleic acid amplification. By integrating this amplification‐free Cas12a activation with multivalent aptamer binding, the assay achieved high sensitivity, detecting as low as 7 CFU/mL.

In parallel, Qian et al. developed an impedimetric electrochemical aptasensor integrated with a statistical machine learning framework for enhanced detection of pathogenic *E. coli* O157:H7 in hydroponic irrigation water.^[^ ^427^ ^]^ This innovative sensor displayed an impressive LOD of 10 CFU/mL. The integration of the statistical machine learning framework significantly improved the prediction of *E. coli* concentrations in complex water matrices, marking a significant advance in rapid, accurate pathogen monitoring for agricultural water management and food safety.

An excellent example to show the evolution of the field of using aptamers to sense heavy metal ions is the sensing of mercury. The interaction between heavy metal ions and biomolecules such as DNA and RNA endows biomaterials with exceptional selectivity, enhancing their capability to recognize and remove heavy metal ions. Aptamers achieve high selectivity toward specific metal ions through a combination of coordination chemistry, conformational adaptability, and evolutionary optimization.^[^ ^18^, ^428^ ^]^ Their nucleobases and phosphate groups can form highly specific coordination bonds with certain metal ions, enabling the formation of metal‐mediated base pairs such as thymine‐Hg^2+^‐thymine (T‐Hg‐T), cytosine‐Ag^+^‐cytosine (C─Ag─C), or guanine‐Pb^2+^‐guanine within G‐quadruplexes. These interactions promote the folding of aptamers into well‐defined 3D structures that accommodate the target ion with a lock‐and‐key‐like fit, while other ions fail to induce the same conformational change. Electrostatic attraction between the negatively charged DNA/RNA backbone and cationic metals provides initial binding, but the geometry and coordination preferences of each ion dictate final selectivity. Furthermore, the SELEX process allows for stringent selection and counter‐selection against competing ions, refining aptamer sequences to achieve exceptional specificity and affinity in complex environments. In essence, aptamers are selective because their unique nucleotide sequences evolved to form specific and stable 3D structures that create a distinct chemical environment precisely matching the size, charge, and coordination preferences of the target metal ion. This molecular precision forms the basis of their outstanding performance in heavy‐metal‐ion sensing.

In 2004, Ono and Togashi pioneered the use of a highly selective oligonucleotide‐based sensor for detecting mercury(II) ions (Hg^2+^) in aqueous solutions, utilizing the unique property of mercury to form stable complexes with thymine‐thymine (T‐T) pairs in DNA duplexes.^[^ ^429^ ^]^ This groundbreaking work introduced a fluorescence‐based sensing mechanism that involved polydeoxyribonucleotides functionalized with a fluorophore and a quencher (Figure 12B). Upon the addition of Hg^2+^ ions, the formation of T‐Hg‐T complexes induced a hairpin structure, leading to quenching of fluorescence. This method achieved a remarkable sensitivity, with a detection limit of 40 nM, significantly surpassing previous small molecular sensors and setting a new standard for the selective detection of Hg^2+^ in the presence of other metal ions.

Based on this technology, in 2008, Li et al. developed two optical methods for detecting Hg^2^⁺ ions using oligonucleotide‐gold nanoparticle hybrids and DNA‐based machines.^[^ ^430^ ^]^ Their approach consisted of the formation of Hg^2^⁺‐bis‐thymine complexes to induce aggregation of AuNPs, resulting in a color change from red to blue. This method allowed the detection of Hg^2^⁺ ions with high sensitivity and selectivity, with a detection limit of 10 nM (2 ppb). The clear red‐to‐blue color change made it suitable for visual detection. They also created a DNA‐based machine that, upon binding Hg^2^⁺ ions, triggered autonomous polymerase activity, resulting in amplified colorimetric detection (Figure 12C). This method had a detection limit of 1 nM (0.2 ppb) and involved two amplification steps, significantly enhancing sensitivity. These findings were important because they offered highly sensitive and selective detection methods for Hg^2^⁺, addressing significant environmental and health concerns.

Since these pioneering developments, the area of aptamer‐based Hg^2+^ detection has experienced incremental progress over the years toward improving the sensitivity of Hg^2+^ aptasensors. Building on earlier work, Huang et al. and Lee et al. further advanced mercury sensing techniques by developing fluorescent gold nanoparticles and DNA‐functionalized gold nanoparticles for colorimetric detection.^[^ ^431^ ^]^ These methods demonstrated the integration of nanomaterials with specific DNA sequences to achieve high sensitivity and selectivity toward Hg^2+^. Subsequently, Wen et al. introduced a highly selective method using α‐hemolysin nanopores and DNA oligomers, marking a significant evolution toward nanotechnology‐based sensors.^[^ ^432^ ^]^ This method stood out for its unparalleled selectivity and low detection limit of 7 nM. Tan et al. demonstrated how the length of aptamer sequences affects the sensitivity of mercury sensors, further enhancing the precision of Hg^2+^ detection in environmental samples.^[^ ^433^ ^]^

A significant advance in this field was made by Li et al., who developed a method to conjugate unmodified diblock DNA to hydrogel nanoparticles and monoliths using a freezing polymerization technique.^[^ ^434^ ^]^ Acrydite‐modified DNA is the most frequently used reagent to prepare DNA‐functionalized hydrogels, but this study showed that unmodified penta‐adenine (A_5_) can reach up to 75% conjugation efficiency, comparable to the ≈83% efficiency of Acrydite‐modified DNA, after eight hours under freezing conditions in polyacrylamide hydrogels. The incorporation efficiency decreased when DNA formed duplexes or folded secondary structures or when the freezing condition was removed, as these factors limit the accessibility of reactive nucleobases. Importantly, such effects can be mitigated by sequence design and maintaining single‐stranded DNA under optimized reaction conditions (such as temperature, ionic strength, and pH). By designing diblock DNA containing an A_5_ block, various functional sequences could be grafted during hydrogel formation. In this design, the A_5_ segment acts as a reactive anchor, forming covalent bonds with acrylamide radicals, thereby enhancing the conjugation efficiency without altering the polymerization mechanism. This work provides a cost‐effective route to attach unmodified DNA to hydrogels while maintaining high functional loading, paving the way for advanced biosensing applications such as ultrasensitive DNA hybridization and Hg^2+^ detection.

Following a similar concept, a method was introduced in 2022 for the efficient detection and removal of Ag^+^ ions, incorporating functional macrocycles and fluorescent molecules into supramolecular polymers.^[^ ^435^ ^]^ This strategy opens new pathways for monitoring pollutants and environmental clean‐up, illustrating the significant potential in pollution control and environmental sustainability.^[^ ^436^, ^437^ ^]^

Despite the advantages of aptasensors for detecting water contaminants, several challenges need to be addressed. These include the potential degradation of nucleic acids by nucleases present in real environmental samples, which can reduce the stability and efficacy of aptamers. Another significant challenge is the specific environmental conditions required for certain types of aptasensors to function effectively. For example, colorimetric aptasensors using AuNPs need precise pH values and high concentrations of NaCl to induce aggregation and visible color change. These stringent conditions can limit their practical applications in diverse water samples where such conditions are not easily met. Moreover, the reusability of aptasensors is often limited because many designs involve immobilized aptamers on nanoparticles or are solution‐based with labelled aptamers, making them single‐use.^[^ ^438^ ^]^ To address this, new materials and designs, such as lab‐on‐a‐chip devices, are being developed to enable automated washing and reuse. Additionally, the sensitivity and specificity of aptasensors can be compromised in complex environmental matrices, leading to false positives or negatives. Enhancing the affinity of aptamers for their targets through chemical modifications, such as adding new nucleobases or using artificial DNA analogues, can help overcome this issue. The development of cooperative binding interactions and SELEX variants also shows promise in improving the performance of aptasensors under native conditions. Despite these challenges, the continuous advancements in aptamer technology, including the integration of high‐throughput sequencing, microfluidics, and bioinformatics, hold great potential for developing more robust, sensitive, and practical aptasensors for environmental monitoring.

### 6.3 Forensic Analysis

Aptamer‐based biosensors hold significant potential for forensic analysis by enabling rapid, sensitive, and specific detection of molecular targets relevant to criminal investigations.^[^ ^402^ ^]^

**Figure 13:**
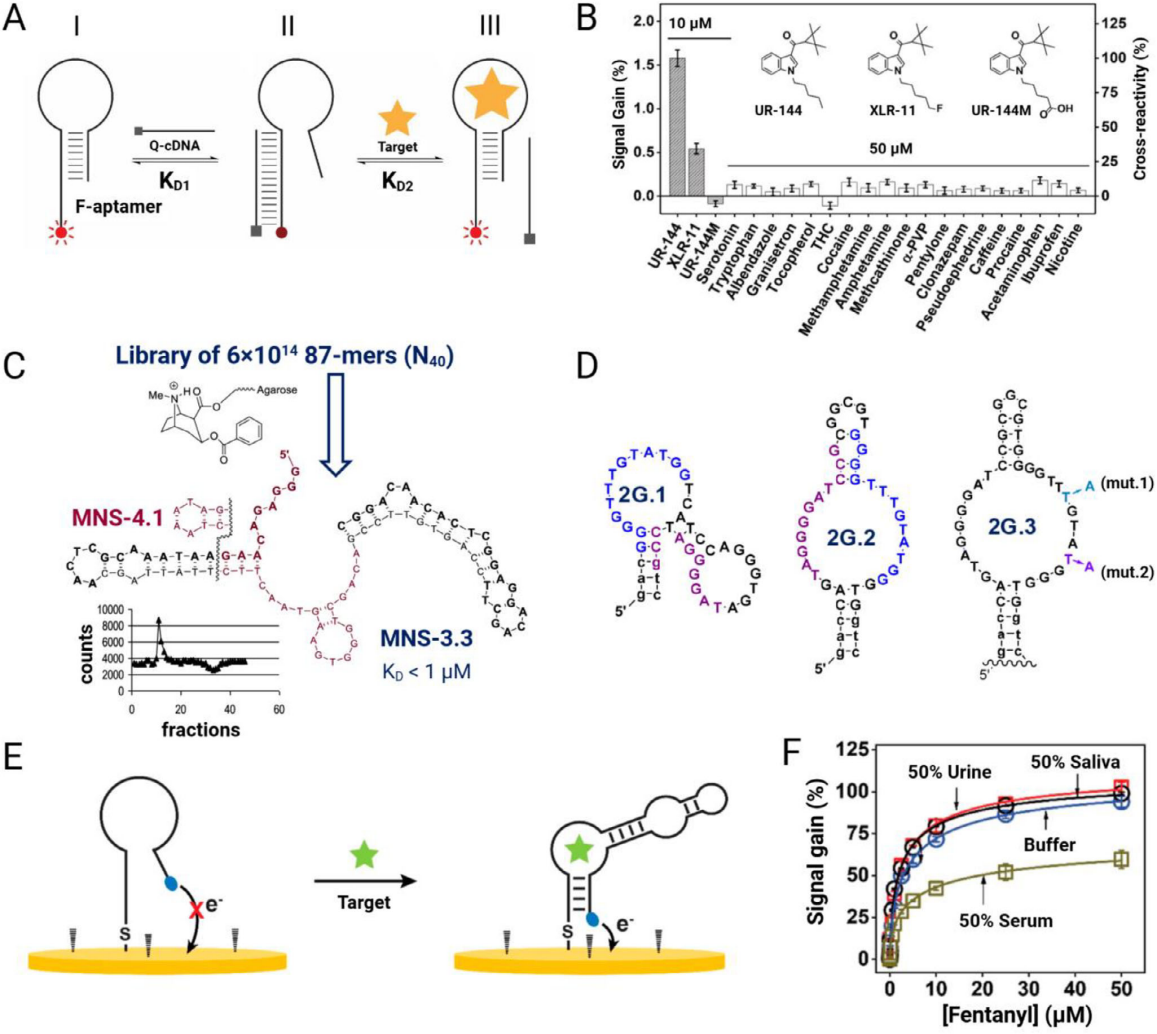
A) Schematic of the strand‐displacement fluorescence assay used for determining the THC‐binding affinity of THC1.2., I) Free F‐THC1.2: When F‐THC1.2 is free in solution, it emits strong fluorescence, II) Hybridization with Q‐cDNA: Addition of Q‐cDNA leads to hybridization, bringing the quencher near the fluorophore, greatly reducing fluorescence emission. *K*_D1_ is determined by titrating different concentrations of Q‐cDNA into a solution of F‐THC1.2. III) Target binding and fluorescence recovery: Titration of THC into F‐THC1.2‐Q‐cDNA complexes causes target binding to the aptamer, resulting in dissociation of Q‐cDNA and recovery of fluorescence. *K*_D2_ is measured during this process, and aptamer target affinity (*K*_D_) is calculated as *K*_D1_/*K*_D2_. B) Signal gains produced by THC, related cannabinoids, and interferents, showing selectivity of the sensor. Adapted with permission.^[^ ^439^ ^]^ Copyright 2021, American Chemical Society. C) Illustration of SELEX‐based cocaine aptamer isolation. Starting with a randomized DNA library, cocaine‐binding aptamers were separated on an agarose column displaying a cocaine analog. Optimization yielded high‐affinity aptamers such as MNS‐4.1. D) Second‐generation cocaine binding aptamers 2G.1 and 2G.2, and a consensus receptor, 2G.3, engineered to represent their common binding pocket core. Adapted with permission.^[^ ^440^ ^]^ Copyright 2024, American Chemical Society. E) Electrochemical aptamer‐based (E‐AB) sensors for fentanyl detection, where target binding induces conformational changes in methylene blue‐labeled aptamers immobilized on gold electrodes, altering the square wave voltammetry signal. F) Calibration curves for fentanyl detection across different biological matrices: buffer (blue), 50% human urine (red), 50% human saliva (black), and 50% human serum (gold). Adapted with permission.^[^ ^441^ ^]^ Copyright 2023, American Chemical Society.

For instance, Yu et al. successfully isolated natural DNA aptamers that exhibit high affinity and specificity for cannabinoids, such as THC, UR‐144, and XLR‐11, from unmodified DNA libraries.^[^ ^439^ ^]^ The detection of their system relies on the fluorescence recovery mechanism, as shown by **Figure** **13**A, where target binding to the aptamer dissociates the quencher‐bound complementary DNA (Q‐cDNA) from the fluorophore‐labeled aptamer, restoring fluorescence proportional to the aptamer‐target affinity, determined as *K*_D_ = *K*_D1_/*K*_D2_. This achievement overcame the challenges presented by the low water solubility and limited functional groups available for aptamer interaction with these compounds. They demonstrated that a well‐designed SELEX procedure with natural DNA libraries can isolate aptamers with high affinity and specificity for challenging small molecules, including cannabinoids such as THC, UR‐144, and XLR‐11. They successfully isolated a DNA aptamer with nanomolar affinity for THC (*K*_D_ of 61 ± 25 nM) and high specificity, overcoming previous difficulties with natural DNA libraries. Additionally, they isolated aptamers with high affinity and cross‐reactivity to UR‐144 and XLR‐11, achieving *K*_D_s of 310 ± 70 nM and 127 ± 32 nM, respectively (Figure 13B). These aptamers maintained excellent specificity against various non‐target interferents. Their work highlights the potential of natural nucleic acid libraries for small‐molecule target aptamer selection and provides a robust workflow for future efforts.

In another work, Yang et al. explored the evolution of DNA aptamers for cocaine detection, moving from cross‐reactive to highly specific, high‐affinity receptors.^[^ ^440^ ^]^ They initially reported MNS‐4.1 (Figure 13C), the first DNA aptamer with micromolar affinity for cocaine, which had broad cross‐reactivity with alkaloids and steroids due to its common structural motif in random oligonucleotide pools. Despite these weaknesses, MNS‐4.1 was widely used in proof‐of‐concept biosensor demonstrations.^[^ ^193^ ^]^ They now report a series of progressively improved DNA aptamers for cocaine, with final optimized receptors achieving low nanomolar affinity and over a thousand‐fold selectivity compared to initial cross‐reactants (Figure 13D). Their optimization process included testing methods to eliminate cross‐reactivities and enhance affinity, achieving properties comparable to reported monoclonal antibody candidates for overdose therapy. Multiple new aptamers share structural motifs with a previously reported serotonin receptor. Further mutagenesis revealed a palindromic, adaptable, broadly cross‐reactive hydrophobic motif that can be rebuilt through mutagenesis and selection into receptors with exceptional affinities and varying specificities.

Canoura et al. reported the successful isolation of fentanyl‐specific aptamers using library‐immobilized SELEX (Figure 13E), highlighting aptamers with nanomolar affinity and exceptional specificity against a wide range of interferents.^[^ ^441^ ^]^ They developed an electrochemical aptamer‐based sensor capable of detecting fentanyl within seconds at clinically relevant concentrations in diluted serum, urine, and saliva (Figure 13F). This breakthrough represents a significant advancement in point‐of‐care diagnostics, offering possibilities for rapid, sensitive, and specific detection of fentanyl in therapeutic monitoring and overdose diagnosis.

More recently, Wang et al. (2024) introduced an aptamer‐based fluorescent sensor for the overly sensitive detection of methamphetamine (METH) using graphene oxide (GO) for fluorescence quenching.^[^ ^442^ ^]^ This sensor demonstrated a low detection limit of 0.78 nM and exhibited excellent selectivity against other common illicit drugs, confirming its potential for practical applications in biological fluid testing. By combining molecular docking to identify aptamer binding sites with GO quenching to enhance sensitivity and selectivity, this approach emerges as a promising tool for on‐site METH detection in saliva and urine.

These examples, along with many others, underline the potential of aptasensors not only in achieving high‐affinity and specificity for a range of targets but also in offering promising solutions for rapid, sensitive, and specific detection in various forensic, food, and environmental applications. A curated selection of aptasensors for non‐medical sensing applications is shown in Table 5. These advances demonstrate a transformative shift toward more efficient and reliable diagnostic methodologies. However, despite their production efficiency, stability, and cost‐effectiveness, aptasensors have not yet been commercially adopted in any area of forensic science.

## 7 Future Directions

Although aptamer engineering has progressed significantly since the pioneering work in the 1990s, there remain numerous challenges (and opportunities) for enhancement of aptamer design. Aptamer selection methods, enhancing aptamer stability and functionality, and advancing the computational design of aptamers are all prerequisite key areas that need to be improved before aptamers' utility and adoption can be expanded.

While there has been incremental progress in developing variations of SELEX for aptamer selection, a common aspect remains that SELEX and its variations are ultimately iterative processes, which hinder the throughput at which new aptamers can be discovered. New methods of screening aptamers in fewer, or even a single selection step, are strongly needed. For example, Singh and colleagues recently published a new method of one‐step aptamer selection using a non‐fouling porous hydrogel,^[^ ^478^ ^]^ which provides a direction for addressing this challenge. Future work will also benefit from microfluidics, droplet‐based screening, and single‐cell platforms that can dramatically accelerate selection and enable parallel discovery against many targets at once. Integration of these technologies with high‐throughput sequencing and AI, including machine learning and large language models for training generative AI, could transform aptamer discovery pipelines into processes rivaling antibody engineering in scale and speed.

Even though aptamers are often rightfully touted for their high stability, there exist opportunities to further improve the stability and functionality of aptamers, mostly through structural means. Due to their inherent nucleic acid composition, aptamers remain vulnerable to nuclease degradation. While we highlighted earlier that nucleic acid modification can protect aptamers from nuclease degradation, if exonuclease protection is desired, circular DNA or RNA aptamers (or “captamers”), such as those found by Liu and colleagues for *Clostridium difficile* detection, can be considered.^[^ ^479^ ^]^ Additionally, the use of DNA for nanofabrication has led to the creation of DNA origami, nanoscale folding to create 2‐ and 3‐D structures from DNA.^[^ ^480^ ^]^ Using both the structural support provided by DNA origami and the target‐specific binding of DNA aptamers, Zhao and colleagues were able to create a DNA origami‐based aptamer nanoarray for anticoagulation in hemodialysis.^[^ ^481^ ^]^ This work highlights the fact that the nucleic acid composition of aptamers provides a unique opportunity for aptamers to work synergistically with other nucleic acid technologies to enhance downstream functionality, and aptamer engineers should keep this in mind when developing new aptamer‐based diagnostic or sensing approaches. In diagnostics, this structural versatility could be harnessed to design multiplexed sensors capable of detecting panels of biomarkers in a single assay. Such multiplexing is particularly relevant for complex diseases, where no single biomarker is sufficient. Furthermore, advances in signal amplification strategies, ranging from catalytic hairpin assemblies to CRISPR‐based readouts, can be paired with aptamers to boost sensitivity to clinically relevant levels.

The recent emergence of AlphaFold three has sparked renewed interest in structural prediction and modeling of biomolecular interactions.^[^ ^128^, ^129^ ^]^ Regrettably, the current AlphaFold three cannot accurately predict aptamer structure and aptamer‐target interactions. The fast‐paced development of current AI tools, including generative AI, has led to the very first AI‐generated drug reaching human clinical trials,^[^ ^482^ ^]^ and has aptamer enthusiasts keenly anticipating our AlphaFold moment. Progress is being made for AI‐generated aptamers,^[^ ^483^, ^484^ ^]^ though further refinement of the current tools is needed before these aptamers see greater utility and adoption. Improving computational pipelines will require combining physics‐based modeling with data‐driven methods and incorporating negative as well as positive selection data to better train algorithms for the rational design of aptamers with desired binding and structural properties. In the near term, hybrid wet lab‐dry lab approaches, where AI designs are rapidly tested through high‐throughput assays, represent a pragmatic route forward. Beyond design, AI may also enable automated analysis of biosensor signals, opening the door to real‐time data interpretation in clinical or field settings.

Equally important are challenges in standardization and reproducibility. Currently, different laboratories report aptamer performance using diverse formats, making it difficult to benchmark across studies. Establishing shared performance standards, databases of validated sequences, and community‐agreed reporting guidelines will be critical to accelerating translation. Finally, the global health potential of aptamers is particularly promising, as their low cost and high stability make them well‐suited for point‐of‐care diagnostics in resource‐limited settings where cold‐chain storage and antibody reagents are not readily available.^[^ ^351^, ^485^, ^486^ ^]^ The lower cost to produce aptamers, compared to the cost to produce antibodies,^[^ ^8^ ^]^ can encourage novel tools for diagnostics and biosensing to utilize the target‐binding abilities of aptamers over antibodies. Portable aptamer‐based sensors, integrated with smartphones or simple readout devices, could democratize access to diagnostics worldwide.

## Author Contributions

J.V.L.N., L.M., and M.T. contributed to the conceptualization of the work. Data curation, formal analysis, validation, and visualization were carried out by J.V.L.N., with support from all authors. Funding was acquired by M.T., with support from L.M. The investigation was performed by J.V.L.N., A.M., C.B., L.M., and M.T. Project administration, resources, and supervision were provided by M.T., with support from L.M. The original draft was written by J.V.L.N., A.M., C.B., and L.M., and all authors contributed to reviewing and editing the manuscript.

## Conflict of Interest

The authors declare no conflict of interest.
